# Experimental evidence that readily diffusible forms of Aβ from Alzheimer’s disease brain have seeding activity

**DOI:** 10.1186/s40478-025-02032-w

**Published:** 2025-05-24

**Authors:** Simin Song, Qianmin Liu, Ruixiang Chen, Ping Chen, Min Tao, Siyao Li, Liping Guo, Xixi Zhu, Yan Liu, Lu Liu, Hiroki Sasaguri, Takashi Saito, Takaomi C. Saido, Dominic M. Walsh, Zhangjin Zhang, Wei Hong

**Affiliations:** 1 Shenzhen Key Laboratory of Neuroimmunomodulation for Neurological Diseases, Shenzhen- Hong Kong Institute of Brain Science, Shenzhen Institute of Advanced Technology, Chinese Academy of Sciences, Shenzhen, 518055 China; 2 Department of Chinese Medicine, The University of Hong Kong-Shenzhen Hospital (HKU-SZH), Shenzhen, 518055 China; 3 Hunan Provincial Key Laboratory of Animal Models and Molecular Medicine, School of Biomedical Sciences, Hunan University, Changsha, 410082 China; 4 Department of Rehabilitation Medicine, Shenzhen Xinhua Hospital, Shenzhen, 518055 China; 5 Laboratory for Proteolytic Neuroscience, RIKEN Center for Brain Science, Wako, 351- 0198 Saitama Japan; 6 Department of Neurocognitive Science, Institute of Brain Science, Graduate School of Medical Sciences, Nagoya City University, Nagoya, 467-8601 Aichi Japan; 7 Laboratory for Neurodegenerative Research, Ann Romney Center for Neurologic Diseases, Brigham and Women’s Hospital, Harvard Medical School, Boston, MA 02115 USA; 8 School of Chinese Medicine, LKS Faculty of Medicine, The University of Hong Kong, Hong Kong, China

**Keywords:** Amyloid β-protein, Cognitive impairment, Gliosis, Neuronal loss, Neurotoxicity, Prion-like spreading, Soluble aggregates

## Abstract

Significant data suggest that cerebral accumulation of the amyloid β-protein (Aβ) plays an initiating role in Alzheimer’s disease (AD), however, Aβ can exist in multiple different forms and it is not clear which of these contribute to the propagation of amyloid or toxicity. When injected into animal models, Aβ-containing homogenates from AD or APP transgenic mouse brain accelerate amyloid pathology, but the nature of the seeding species remain ill-defined. In this study, we took advantage of well-characterized brain extracts from human AD cases and *App*^*NL−F/NL−F*^ mice, to assess the seeding activity of diffusible forms of Aβ. Extracts containing readily diffusible forms of Aβ, (which we refer to as *S extracts*) are obtained by soaking tissue slices in physiological buffer and removing large non-diffusing material by centrifugation. Such AD brain *S extracts* are potent neuritotoxins and contain a broad range of different sized forms Aβ. When tested at approximately ten months after a single intracerebral injection of AD brain *S extract*, *App*^*NL−F/NL−F*^ mice exhibited a significant perturbation of learned behavior, together with accelerated cerebral amyloid deposition, microgliosis, astrocytosis, neuronal dystrophy and synaptic loss. Importantly, inoculation of *App*^*NL−F/NL−F*^ mice with *S extract* from a human control brain altered neither the memory of learned behavior nor the appearance of amyloid and associated pathologies. These results indicate that diffusible forms of Aβ derived from AD brain can readily induce aggregation of endogenous Aβ and accelerate negative outcomes associated with Aβ accumulation.

**Supplementary Information:**

The online version contains supplementary material available at 10.1186/s40478-025-02032-w.

## Introduction

Growing evidence from animal models and human studies has implicated the propagation and spreading of protein assemblies in the pathogenesis of Alzheimer’s disease (AD), Parkinson’s disease and other neurodegenerative disorders [1]. A common and long-standing question of interest for proteins associated with neurodegeneration is how their aggregation and deposition relates to disease progression [2]. Compelling genetic and biomarker evidences have shown that cerebral accumulation of the amyloid β-protein (Aβ) initiates complex molecular and cellular cascades that lead to AD pathogenesis [3, 4]. The transmission of Aβ aggregation has been examined extensively in rodents and primates [5–7]. Intracerebral inoculation with Aβ-containing brain homogenates or extracts can accelerate Aβ deposition in genetically modified mouse models, Wistar rats and non-human primates [8, 9]. Iatrogenic cerebral Aβ pathology in humans has been detected following administration of cadaver-sourced human growth hormone or dura mater graft containing Aβ [10, 11]. Such observations emphasize the need to prevent accidental transmissions of disease-related protein assemblies via medical and surgical procedures, and that stopping the propagation and spreading of pathogenic Aβ may be a promising way to slow down disease progression. A recent animal study showed that acute targeting of Aβ seeds before Aβ deposition becomes detectable reduces Alzheimer-like pathology later in life [12].

Given the widespread interest in Aβ transmission, significant efforts have been made to characterize the seeding capacity of Aβ isolated from human or animal specimens [13–15]. Heretofore most studies have utilized crude brain homogenates in which the predominant Aβ species are insoluble aggregates. Unfortunately, the methods for isolating Aβ-containing inocula from brain tissue vary widely between laboratories and in publications from the same group. In most cases cortical tissue was mechanically homogenized in aqueous buffer, diluted and directly. Only in a few cases were brain homogenates ultracentrifuged and the supernatant used [11, 16, 17]. Sonication of brain extracts, intended to break up large aggregates, prior to intracerebral injections led to acceleration and more widespread distribution of amyloid pathology [8, 14], thus hinting that smaller more readily diffusible species are more effective seeds. Consequently, the definition of Aβ species capable of seeding remains primitive.

In an effort to address this important issue, we utilized a recently developed gentle extraction protocol. This method involves soaking minced AD brain tissue in aqueous buffer to allow readily diffusible material to enter the buffer. Next sequential centrifugation removes cellular debris and then large macromolecular assemblies, the final supernatant is referred to as the *S extract*. Unlike homogenization, this soaking protocol minimizes the physical disruption of larger aggregates, such that the quantity of Aβ present in *S extract* is only around one fifth of that in homogenates [18, 19]. We and others have previously shown that soluble Aβ aggregates directly isolated from human AD brains disrupt synaptic plasticity and neurite integrity, induce tau phosphorylation, impair hippocampal long-term potentiation (LTP), and induce neuronal hyperactivation and cognitive decline [19–23]. Importantly the readily diffusible Aβ species present in *S extract* account for essentially all bioactivity present in extracts produced by homogenization [19]. However, it is not known if diffusible Aβ aggregates extracted by the gentle soaking method are capable of seeding Aβ aggregation in vivo.

In this study, we inoculated *App*^*NL−F/NL−F*^ mice with well-characterized *S extracts* from AD patients or a healthy control (HC), and assessed amyloid accumulation and the ability of mice to recall learned behavior compared with vehicle injected mice. Prior to inoculation, brain extracts were characterized for their Aβ content, assembly size and effects on neurite integrity when applied to iPSC-derived neurons (iNs). As seen previously, *S extract* from human AD brain contained Aβ with a broad range of assembly sizes and when applied to iNs, *S extract* reduced neurite length and number in a manner that requires Aβ. Significant decline of working memory and spatial learning and memory in *App*^*NL−F/NL−F*^ mice was induced at 300–315 days post-inoculation (dpi) by a single inoculation of *S extracts* from AD brains, but not HC brain. Thereafter, we demonstrate that at 316–320 dpi the AD *S extracts* accelerated amyloid deposition in cortex and hippocampus and this was accompanied with microgliosis and astrocytosis. Moreover, severe neuronal dystrophy and synaptic loss surrounding amyloid plaques were also observed in mice inoculated with AD *S extracts*, while only modest effects were detected in animals that received vehicle or HC *S extract*. Collectively, these results indicate that diffusible Aβ species extracted from AD brains have both acute toxicity and seeding activity, and that the latter can be experimentally transmitted.

## Materials and methods

### Reagents and chemicals

Aβ1–40 and Aβ1–42 peptides were synthesized and purified using Reverse-Phase High-Performance Liquid Chromatography (RP-HPLC) by China Peptides Co., Ltd (Shanghai, China). Peptide mass and purity (> 95%) were confirmed by RP-HPLC and electrospray/ion trap mass spectrometry. Peptide standards for Aβ immunoassays were prepared as described previously [19]. Briefly, lyophilized Aβ stocks were denatured in 7 M guanidium-HCl (GuHCl) solution at room temperature overnight and monomers separated by size-exclusion chromatography. Peptide was then diluted to 10 µg/ml, aliquoted and stored frozen at -80 °C until use. Gel filtration standards were purchased from Bio-Rad (Hercules, CA). All other chemicals and reagents were of the highest purity available and unless indicated otherwise were obtained from Sigma-Aldrich (St. Louis, MO).

### Antibodies

The antibodies used in this study and their sources are described in Table 1.

**Table 1:** Antibodies used in this study and their sources

Antibody (target)	Type	Assay	Conc./dilution	Source/Reference
266 (Aβ16–23)	Mouse	ELISA	3 µg/ml	Elan [54]
2G3 (Aβx-40)	Mouse	ELISA	0.2 µg/ml	Elan [55]
21F12 (Aβx-42)	Mouse	ELISA	0.4 µg/ml	Elan [55]
S97 (Pan anti-Aβ)	Rabbit	ICC	1:1000	Walsh Lab [35]
6E10 (Aβ6–10)	Mouse	ICC	1:500	Biolegend [56]
NCNP24 (Iba1)	Rabbit	ICC	1:500	Wako, Cat #019-9741
GA5 (GFAP)	Mouse	ICC	1:500	Cell signaling, Cat #3670S
EPR23531-50 (Synapsin-1)	Rabbit	ICC	1:500	Abcam, Cat #ab254349
RM1010 (MAP2)	Rabbit	ICC	1:500	Abcam, Cat #ab32454
Alexa Fluor 488 anti-mouse	Goat	ICC	1:500	Abcam, Cat #ab150113
Alexa Fluor 488 anti-rabbit	Goat	ICC	1:500	Abcam, Cat #ab150077
Alexa Fluor 594 anti-mouse	Goat	ICC	1:500	Abcam, Cat #ab150116
Alexa Fluor 594 anti-rabbit	Goat	ICC	1:500	Abcam, Cat #ab150080

### Animals

All animal experiments this study were performed in accordance with institutional animal care and use guidelines, and were approved by the Institutional Animal Care and Use Committees (IACUC) at the Shenzhen Institute of Advanced Technology, Chinese Academy of Sciences (approved case ID: SIAT-IACUC-20220801-NS-NJBZX-HW-A2091-01). We used homozygous *App*^*NL−F/NL−F*^ mice that express APP bearing the Swedish (KM670/671NL) and Beyreuther/Iberian (I716F) mutations, and in which the Aβ domain has been humanized [24]. Mice were speed-backcrossed and maintained on an inbred C57BL/6J background to remove potential extraneous mutations, and used as homozygotes. Wild type (WT) C57BL/6J mice were purchased from Guangdong Vital River Laboratory Animal Technology Co., Ltd. Mouse genotype was confirmed by polymerase chain reaction (PCR) of tail-punch DNA, and mice were uniquely identified by ear tag. Only male mice were used in the current study to minimize variability. All mice were housed in standard mouse cages under a 12-h dark/12-h light cycle and constant temperature and humidity. Food and water were provided ad libitum.

### Preparation of *S extracts* from human brain

**Fig. 1:**
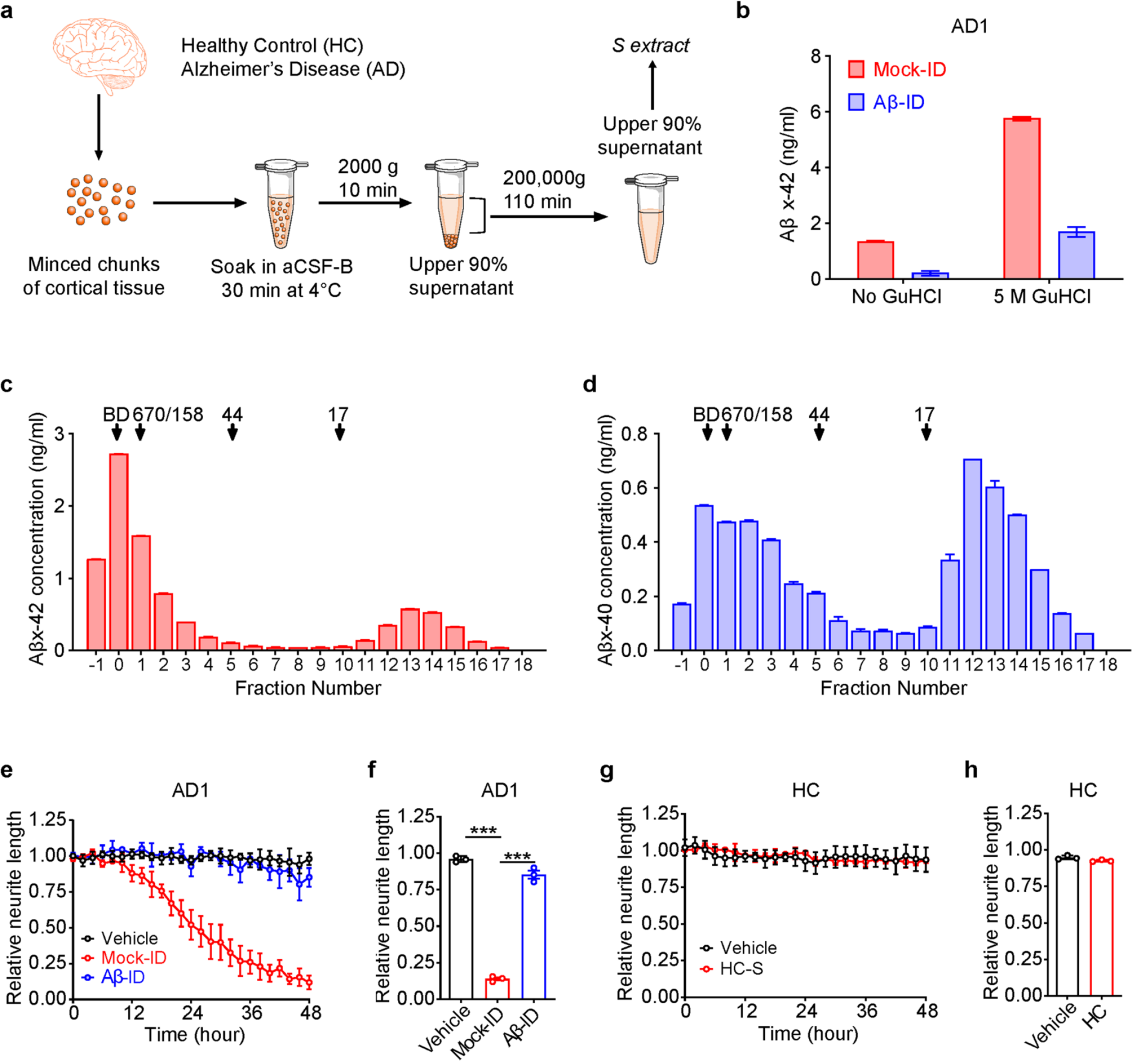
Bioactive *S extract* of human AD brain contains both high and low molecular weight Aβ species. (**a**) Schematic showing the protocol used to prepare *S extracts* of human brains. (**b**) Aβx − 42 immunoassay quantification of Aβ in AD1 *S extract* after immunodepletion with S97 (Aβ-ID) or preimmune serum (Mock-ID). (**c**,** d**) *S extract* from AD1 was fractionated using a Superdex 75 size exclusion column eluted with 50 mM ammonium bicarbonate, pH 8.5. Fractions were lyophilized, denatured with GuHCl and analyzed using the MSD-based x-42 and x-40 assay. Elution of gel filtration standards is indicated by downward pointing arrows labeled 17, 44, 158, and 670 (in kDa). Fraction 0 indicates the peak fraction in which Blue dextran (BD) eluted. (**e**,** g**) Human iPSC-derived neurons (iNs) were treated with vehicle, Mock-ID’d or Aβ-ID’ed AD1 *S extract*, HC *S extract* and imaged for 48 h. NeuroTrack-identified neurite length was calculated relative to baseline collected 6 h prior to addition of sample. All samples were tested at 1:4 dilution. Values are the average of triplicate wells ± SEM. (**g**,** h**) Histogram plots of normalized neurite length are derived from the last 3 recordings the traces shown in **c** and **e**. Values are the average of triplicate wells ± SEM. Significant difference is denoted as ***p < 0.001

Human specimens were obtained from the Massachusetts ADRC Neuropathology Core, Massachusetts General Hospital and used in accordance with the Partners Institutional Review Board (Protocol: Walsh BWH 2011). Frozen cortical tissues used in this study were obtained from two individuals (referred to as AD1 and AD2) who died with AD and one individual who died free of AD (designated HC). AD1 was a 69-year-old female and AD2 was an 83-year-old male both died with end-stage AD, and met clinical (CERAD) and pathological criteria for AD (Braak VI). AD1 had a postmortem interval (PMI) of 4 h and AD2 a PMI of 12 h. HC was a 92-year-old male who died free of AD symptoms and pathology with a PMI of 18 h. Aqueous brain extracts were prepared as described previously [19]. Briefly, gray matter was dissected and minced into small chunks using a McIlwain chopper, and then soaked in five volumes of ice-cold base artificial cerebrospinal fluid (aCSF) plus protease inhibitors at 4 °C for 30 min with gentle side-to-side mixing. Samples were centrifuged at 2,000 g at 4 °C for 10 min to remove cellular debris. Then the upper 90% of the supernatant was collected and centrifuged at 200,000 g for 110 min at 4 °C. The upper 90% of this second supernatant designated as *S extract* was removed and dialyzed to remove small potentially bioactive molecules (Fig. 1a). Briefly, *S extracts* were dialyzed (Slide-A-Lyzer™ G2 Dialysis Cassettes, 2 K MWCO, Fisher Scientific) against 100-fold excess of fresh aCSF buffer with gentle agitation at 4 °C. Buffer was changed three times over a 72 h period. Dialyzed *S extracts* were aliquoted and stored at -80 °C until use. A portion of each *S extract* underwent 3 rounds of incubations with either: (i) the pan anti-Aβ antibody S97 to immunodeplete the sample of Aβ (Aβ-ID), or (ii) pre-immune rabbit serum to provide a non-immunodepleted control (Mock-ID). Thereafter, the samples were stored at -80 °C, thawed when required, and used immediately.

### Measurements of Aβx-40 and Aβx-42 by MSD immunoassays

The Aβx-40 and Aβx-42 immunoassays preferentially detect Aβ monomers ending at Val40 and Ala42, respectively. Both assays use monoclonal antibody m266 (3 µg/ml) for capture. The x-40 assay uses biotinylated 2G3 (0.2 µg/ml) for detection and the x-42 assay uses biotinylated 21F12 (0.4 µg/ml) for detection. Previous studies showed that incubation of brain extracts with GuHCl effectively dissociates Aβ aggregates and allows detection of monomer by the x-40 and x-42 assays [25]. Brain extracts from AD1 and AD2 were analyzed with and without pre-incubation in 5 M GuHCl. Briefly, 20 µl of sample was incubated with 50 µl of 7 M GuHCl at 4 °C overnight. Thereafter samples were diluted 1:10 with diluent so that the GuHCl concentration was 0.5 M. SEC-isolated Aβ1–40 and Aβ1–42 monomers were used as standards and prepared with or without 0.5 M GuHCl to match the buffer composition of loaded samples. The assays were performed using the Meso Scale Discovery (MSD) platform and reagents from Meso Scale (Rockville, MD). Samples, standards and blanks were loaded in triplicate and analyzed as described previously [19].

### Size exclusion chromatography (SEC)

Samples were chromatographed on a Superdex 75 10/300 GL column eluted with 50 mM ammonium bicarbonate, pH 8.5 at 0.5 ml/min. Prior to chromatographing samples, the column was calibrated using Blue dextran and gel filtration standards. The peak fraction in which Blue dextran eluted was designated as fraction zero. Immediately prior to use, dialyzed *S extract*s were thawed, vortex mixed and then spun at 12,000 rpm on an Eppendorf centrifuge for 10 min. The upper 0.95 ml of sample was removed and loaded onto the SEC column and 0.5 ml fractions collected and lyophilized. To enable detection of Aβ with different aggregation states, lyophilizates were reconstituted in 50 µl of 5 M GuHCl and incubated at 4 °C overnight. Thereafter, samples were diluted 1:10 with assay diluent and analyzed using the MSD-based Aβx-42 and x-40 immunoassays.

### Preparation of human iPSC induced neurons (iNs)

Human iPSC line DYR0100 (derived from the foreskin fibroblast cell line SCRC-1041) was kindly provided by Stem Cell Bank, Chinese Academy of Sciences. Plasmids pTet-O-Ngn2-puro (Addgene plasmid #52047) and Tet-O-FUW-EGFP (Addgene plasmid #30130) were gifts from Marius Wernig [26, 27]. Plasmid FUdeltaGW-rtTA (Addgene plasmid #1978) was a gift from Konrad Hochedlinger [28]. All the lentiviruses used in this study were packaged and titers determined by Taitool (Shanghai Taitool Bioscience Co., Ltd, China). Endogenous Neurogenin 2 (Ngn2), Green Fluorescent Protein (GFP) and reverse tetracycline transcriptional activator (rtTA) genes were introduced by lentivirus infection. Neuronal differentiation was performed via doxycycline induced NGN2 system as previously described [19, 26]. Briefly, doxycycline is added on “iN day 1” at a concentration of 2 µg/ml to switch on the expression of NGN2. On iN day 2 puromycin is added at 10 mg/ml and is maintained in the media at all times thereafter. On iN day 4, cells were frozen down or directly plated on Greiner 96-well microclear plates pre-coated with a combination of Matrigel and poly-lysine which can improve neuronal differentiation [29]. Cells were then maintained in media consisting of Neurobasal medium (Gibco), Glutamax, 20% Dextrose, MEM-NEAA and B27, and BDNF, CNTF, GDNF (PeproTech, Rocky Hill, NJ) each at a concentration of 10 ng/ml.

### Live-cell imaging to assess the neuritotoxic activity of *S extracts*

Prior studies indicated that iNs require 2–3 weeks to reach maturation [29, 30]. In this study, iNs at day 21 were used to investigate the effects of AD brain extracts on neuritic integrity. Brain extracts were thawed on ice and exchanged into neurobasal medium supplemented with B27/Glutamax using a G25 MidiTrap desalting column (GE Healthcare). Typically, desalted brain extracts were added to iNs at a dilution ratio of 1:4, whereas higher dilution factors were used for dose-dependent tests. iNs were monitored continuously using the IncuCyte S3^®^ live-cell imaging system (Essen Bioscience, Ann Arbor, MI). Phase contrast images were collected at 2 h intervals for a total of 2–4 days and analyzed using the NeuroTrack analysis job. Neurite processes were automatically defined by the analysis and data subsequently acquired for neurite length.

### Intracerebral injections of human brain extract

*S extract*s from AD1, AD2 and HC were thawed on ice, exchanged into Dulbecco’s Phosphate-Buffered Saline (D-PBS) lacking Ca^2+^ or Mg^2+^ ions using a G25 MidiTrap desalting column (GE Healthcare) and used for inoculation. Intracerebral inoculation was performed as described previously [11]. Mice (male, 8 weeks old) were randomly assigned to experimental groups, anaesthetized with isoflurane and intracerebrally inoculated into the right hemisphere in the parietal region with 7 µl of samples from AD1, AD2, HC or vehicle (referred to D-PBS in this study).

### Y-maze

At 300 dpi, mice were tested for short-term working memory in Y-maze using a standard protocol [31]. Testing was carried out using a Y-shaped maze with three light-colored, opaque arms orientated at 120° angles from each other. In each test, an individual mouse was introduced at a particular position on the maze and allowed to explore the arms freely. Spontaneous alternation behavior during a 5 min test period was recorded. An alternation was defined as consecutive entries into all three arms. The number of total arm entries and alternations are used to calculate the percentage of the alternation behavior. A high percentage of alternations is taken as a good working memory as this indicates that the mouse has recalled which arms it has already visited.

### Barnes maze

The Barnes maze test was performed at 305 dpi (5 days after the completion of Y-maze task) to assess long-term spatial memory function [32]. A typical Barnes maze consists of an elevated circular platform with 40 evenly-spaced holes (5 cm diameter) around the perimeter. An escape tunnel is mounted underneath one hole while the remaining 39 holes are left empty. In each habituation trial (305–306 dpi), mouse was allowed to explore on the platform freely for 5 min without the escape tunnel, and habituated to the escape tunnel for 2 min on two consecutive days. During the course of training (307–311 dpi), mouse was covered by a nontransparent cylinder placed in the center of the maze for 15 s. The cylinder was then gently removed to allow mouse to explore the maze for 180 s until the target hole was found. The primary latency that mice took to find the target hole was documented for each trial. Three days after the last session of acquisition training, a probe trial was administered to assess spatial reference memory in the absence of the escape tunnel. The probe trial was performed in a similar manner to the acquisition trials except that the duration of the probe trial was 90 s. The primary latency to find the target hole and duration time in the target quadrant were analyzed by ANY-maze software (Global Biotech Inc. Shanghai, China).

### Tissue collection and immunofluorescence staining

Upon completion of Y-maze and Barnes maze assessments (316 dpi), mice were anesthetized with isoflurane and perfused with ice-cold Phosphate-Buffered Saline (PBS) followed by PBS containing 4% paraformaldehyde (PFA). Brains were dissected, fixed in PBS containing 4% PFA for 72 h at 4 °C, and dehydrated in 30% sucrose. All mice used in this study were sacrificed and brain tissue collected within 5 days (from 316 to 320 dpi). Thereafter, brains were embedded by optimal cutting temperature (OCT) compound (Tissue-Tek, Sakura Finetek, USA) and processed for serial sections (35 μm) using a freezing microtome (Leica CM1950, Leica, Germany). Antigen retrieval was performed by incubating slides in 70% formic acid for 20 min at room temperature. Brain sections were washed 3 × 5 min with PBS, then blocked with 5% Bovine Serum Albumin (BSA) and 0.1% Triton X-100 in PBS for 1.5 h at room temperature. Primary antibodies were diluted in PBS containing 1.25% BSA and 0.1% Triton X-100, applied to brain sections and incubated overnight at 4 °C. Thereafter, brain sections were washed 3 × 5 min with PBS containing 0.1% Triton X-100. Bound antibodies were detected by the addition of the appropriate Alexa Fluor conjugated secondary antibodies (diluted in PBS containing 1.25% BSA and 0.1% Triton X-100) for 2 h at room temperature. After washing with PBS, sections were stained with 6-diamidino-2-phenylindole (DAPI) for 10 min, washed 3 times in PBS and then mounted on glass slides. Fluorescent images were captured using a confocal microscope (Zeiss LSM 900) or slide scanner (Olympus, VS120). To visualize dense plaques, stained sections were incubated in 500 µM of Thioflavin-S (ThS) dissolved in 50% ethanol for 7 min at room temperature, washed twice with 50% ethanol and 3 times with PBS prior to image acquisition. Quantitative analysis of images was performed using ImageJ software. To determine the number and area of Aβ plaques, as well as the ionized Calcium Binding Adaptor Molecule 1 (Iba1)^+^, Glial fibrillary acidic protein (GFAP)^+^, Synapsin I (SYN1)^+^ and Microtubule Associated Protein 2 (MAP2)^+^ area or intensity in the prefrontal cortex and hippocampus regions, data was acquired from at least 3 slides of each mouse and at least 7 mice per group.

### Statistical analysis

Statistical analysis of behavioral results was performed by SPSS25.0 software (SPSS Inc., Chicago, IL, USA). IncuCyte and immunostaining data was analyzed by GraphPad10 software (GraphPad Software Inc., La Jolla, CA, USA). All data are presented as means ± standard errors unless otherwise stated. Shapiro-Wilk test was used to test the normality. For multiple comparisons, normally distributed data were analyzed using one-way analysis of variance (ANOVA) with Tukey’s *post hoc* test, while nonparametric data were analyzed with the Kruskal-Wallis test followed by Dunn’s *post hoc* test. For comparison between two groups, normally distributed data were analyzed by unpaired two-tailed Student’s *t* tests, and nonparametric data were analyzed using Mann-Whitney tests. The significance threshold was set as *p* < 0.05 and significance levels are indicated by *p < 0.05, **p < 0.01 and ***p < 0.001. The inbred C57BL/6J WT mice were from Guangdong Vital River Laboratory Animal Technology Co., Ltd and therefore are not a perfect match for *App*^*NL−F/NL−F*^. The principal biological comparisons are AD inoculated mice versus control and vehicle injected animals. These mice were used to provide a comparative baseline and the comparison between WT mice and *App*^*NL−F/NL−F*^ mice was analyzed separately. To statistically analyze pathological changes in the hippocampus, data encompassing the entire hippocampal region were used.

## Results

We and others have shown that extracts of human cortical tissue prepared by a gentle soaking method impair neurite integrity and synaptic plasticity in a manner that requires Aβ [19, 33]. We previously demonstrated that this bioactive fraction contains a range of different sized Aβ species [18, 19] which others have reported to include protofibrils [34]. Here we investigated whether the diffusible Aβ species present in *S extracts* are capable of seeding amyloid deposition in brains.

### Bioactive aqueous extracts of human AD brains contain both high and low molecular weight Aβ species

Aqueous extracts of human cortex were prepared by soaking minced tissue in aCSF-B for 30 min followed by sequential centrifugation (Fig. 1a). Under native conditions the monomer preferring x-42 immunoassay readily detected signal in AD1 *S extract*, and pre-treatment of AD1 *S extract* with aggregate-disrupting denaturant (5 M GuHCl) dramatically increased the amount of Aβ detected (Fig. 1b). These results are consistent with prior studies in which demonstrated that 5 M GuHCl allows denaturation of Aβ aggregates into monomers [19, 25]. AD2 *S extract* contained levels of Aβx-42 comparable to AD1, but Aβx-42 was not detected in the HC *S extract* (Supplementary Fig. 1e). To assess the presence and abundance of different sized Aβ species we used SEC to fractionate the AD1 *S extract* and then denatured the fractions to maximize detection by our monomer-preferring Aβx-40 and x-42 immunoassays [19]. Aβ was detected from the void volume of the column to very low molecular weight (Fig. 1c and **d**). Most Aβ detected by the Aβx-42 assay eluted in one of two broad peaks. The first spanned fractions #-1 to 2, which are indicative of assemblies larger than globular proteins of ~ 158 kDa (Fig. 1c). A second less abundant peak detected in fractions #12 to 15 is consistent with true Aβx-42 monomers (Fig. 1c). In addition to the high molecular weight and monomer peaks small amounts of Aβx-42 of intermediate molecular weights were also detected. The x-40 assay detected signal in the same fractions in which x-42 was detected, but with some notable differences (Fig. 1d). First, the overall abundance of Aβx-40 was approximately half that of Aβx-42. Second, the Aβx-40 high molecular weight peak was much broader and included material of molecular weights down to ~ 44 kDa. Third, the amount of Aβx-40 in the monomer peak relative to the high molecular weight peak was higher than for Aβx-42. Overall, these data indicate that the AD1 *S extract* contains a complex mixture of different Aβ alloforms and assembly sizes.

To assess their bioactivity, we utilized IncuCyte live-cell imaging of iPSC-derived human neurons (iNs). *S extract*s from both AD1 and AD2 caused a time- and dose-dependent loss of neuritic complexity, while HC *S extract* had no effect (Fig. 1e-h, Supplementary Fig. 1a-d). Immunodepletion of Aβ from the AD1 *S extract* prevented the reduction of neurite length caused by AD1, indicating the toxic effect was Aβ-dependent (Fig. 1e-f). Although the *S extract*s of AD1 and AD2 contained similar levels of Aβx-42, AD1 was significantly more potent than AD2 (Supplementary Fig. 1). These results are consistent with the prior observations that the amount of Aβx-42 does not predict Aβ-dependent bioactivity [35].

### Intracerebral inoculation of *App*^*NL−F/NL−F*^ mice with AD-*S extracts* impairs both working memory and spatial learning and memory

Next, we examined if AD-*S extract*s are capable of disrupting learned behavior in *App*^*NL−F/NL−F*^ mice and how this might relate to seeding of amyloid deposition. *App*^*NL−F/NL−F*^ mice are knock-in mice which express partially humanized APP within the endogenous murine *APP* locus and evince an age-dependent accumulation of amyloid and deterioration in the performance of various learned behaviors [24, 36]. *App*^*NL−F/NL−F*^ mice express APP bearing a fully humanized Aβ sequence together with two AD causing mutations at either side of the Aβ domain - the Swedish (KM670/671NL) and Beyreuther/Iberian (I716F) mutations [24, 37]. These mice produce more Aβx-42 than Aβx-40 with age and are a useful model for seeding as the Aβ seeds in AD brain extracts are mainly composed of x-42 species. Previous study showed that cognitive impairment in *App*^*NL−F/NL−F*^ mice emerged at 18 months old as measured by Y-maze task, while no significant deficit was seen on the Morris water maze [24]. Thus, these mice offer a long time window to evaluate the potential for *S extracts* to accelerate cognitive and histological phenotypes. In these experiments the principal biological comparisons are between AD *S extract* inoculated mice versus HC *S extract* and vehicle injected animals.

**Fig. 2:**
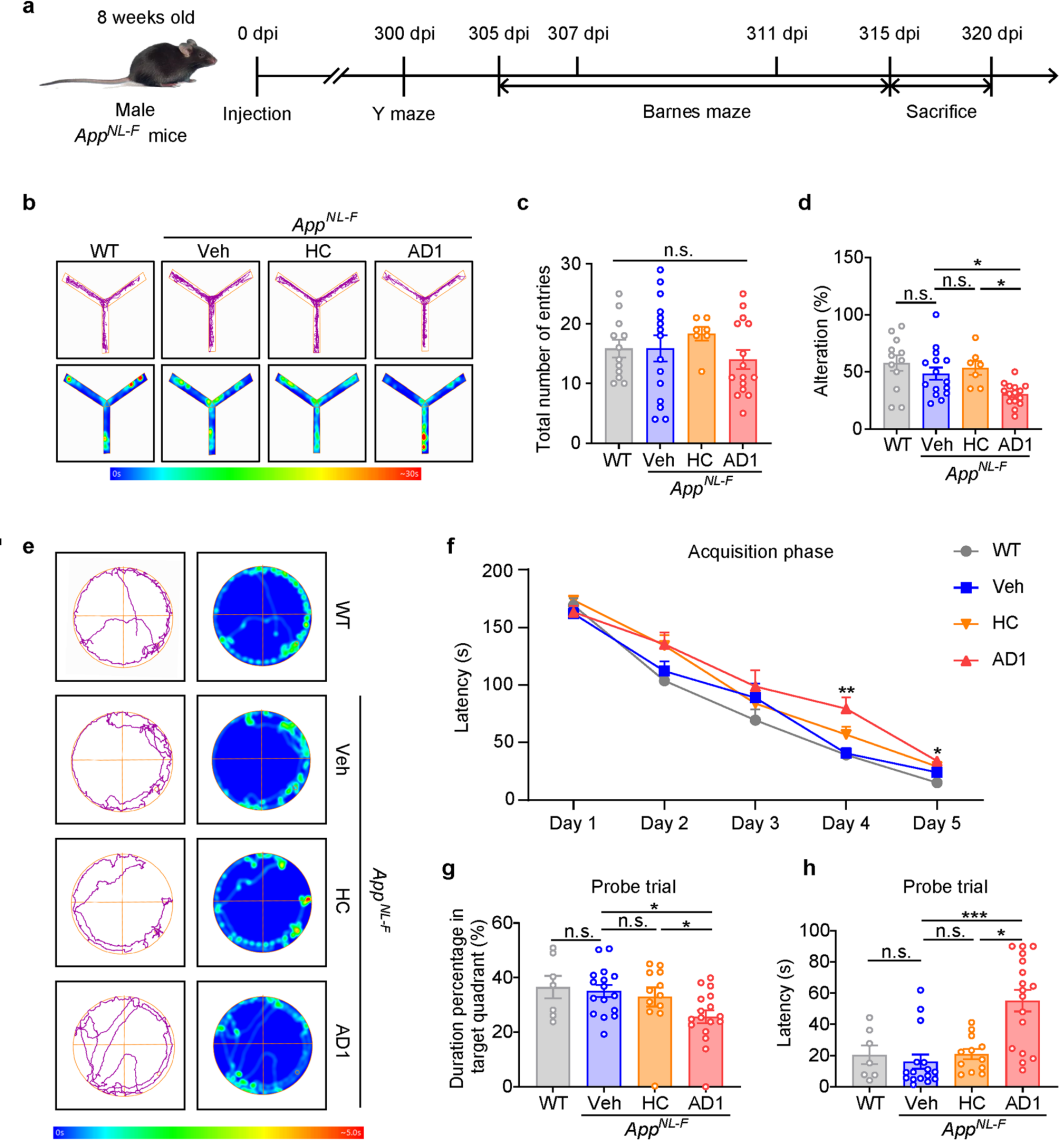
Intracerebral inoculation of *App*^*NL−F/NL−F*^ mice with AD1 *S extract* impairs s both working memory and spatial learning and memory. (**a**) Schematic showing the timeline of inoculation, behavioral tests and tissue collection. Working memory was measured by Y-maze at 300 dpi and spatial learning memory was assessed by Barnes-maze during 305–315 dpi. The latter included a habituation trial (305–306 dpi), an acquisition phase (307–311 dpi), and a training probe trial (315 dpi). (**b**) Representative trajectories and heat maps of mice in Y-maze task. (**c**) Total number of mice entries into the three arms of the Y-maze: WT (*n* = 12), Veh (*n* = 15), HC (*n* = 7) and AD1 (*n* = 15), *p* > 0.05. (**d**) The percentage of correct alterations in Y-maze: WT (*n* = 12), Veh (*n* = 15), HC (*n* = 7) and AD1 (*n* = 15). AD1 vs. Veh, *p* = 0.013; AD1 vs. HC, *p* = 0.011; F = 6.630, one-way ANOVA. (**e**) Representative trajectories and heat maps of mice in Barnes-maze. (**f**) The latency to find the escape tunnel during the 5 days acquisition phase in Barnes-maze: WT (*n* = 7), Veh (*n* = 16), HC (*n* = 12) and AD1 (*n* = 17). Day 4, AD1 vs. Veh, *p* = 0.001; Day 5, AD1 vs. Veh, *p* = 0.036. (**g**) The percentage of time spent in the target quadrant during the probe test in Barnes-maze: WT (*n* = 7), Veh (*n* = 16), HC (*n* = 12) and AD1 (*n* = 17). AD1 vs. Veh, *p* = 0.039; AD1 vs. HC, *p* = 0.019; Kruskal–Wallis test. (**h**) The primary latency to find the escape tunnel during the probe test in Barnes-maze: WT (*n* = 7), Veh (*n* = 16), HC (*n* = 12) and AD1 (*n* = 17). AD1 vs. Veh, *p* < 0.001; AD1 vs. HC, *p* = 0.024; Kruskal–Wallis test. Significant differences are denoted as *p < 0.05, **p < 0.01, and ***p < 0.001. n.s. denotes not significant. Values are shown as mean ± SEM

In preliminary time-course experiments inoculation of male *App*^*NL−F/NL−F*^ with AD *S extract* manifested changes in learned behavior at 300 dpi (data not shown), hence this was the interval we chose for our larger study to which we incorporated appropriate control treatments. Here, male *App*^*NL−F/NL−F*^ mice received intracerebral injections of *S extracts* (AD1 or HC) at 8 weeks age and their performance on the Y-maze and Barnes maze was tested approximately 10 months later. Y-maze was performed on 300 dpi and after a 5 days’ rest, training in the Barnes maze was initiated (i.e., 305 dpi) (Fig. 2a). Mice that received vehicle or HC *S extract* performed similarly on the Y-maze as did WT mice. In contrast, significant reduction of alteration was observed in the mice that received AD1 *S extract* (Fig. 2b-d). Only a small number of AD2 injected mice were tested on the Y-maze and these performed similarly to vehicle injected mice (Supplementary Fig. 2a-c).

During the last two days of the acquisition phase of the Barnes maze, mice which received AD1 *S extract* showed a significant increase in the latency to locate the escape tunnel than mice injected with vehicle, indicating impairment of learning memory (Fig. 2f). In the probe phase, mice which received AD1 *S extract* spent significantly less time in the target quadrant than vehicle or HC-*S extract* injected mice and had a longer latency to locate the escape tunnel (Fig. 2e, g and **h**). Relative to vehicle injected mice, animals inoculated with AD2 *S extract* had a progressive increase of latency to find the escape tunnel during the acquisition phase of Barnes maze (Supplementary Fig. 2e). In the probe phase, AD2 injected mice also spent significantly less time in the target quadrant than vehicle injected mice and had a longer latency to locate the escape tunnel (Supplementary Fig. 2d,** f** and **g**). These data show that intracerebral inoculation of AD *S extracts* induce significant impairment of spatial reference memory at an age when vehicle and HC *S extract* injected mice performed at level comparable to WT controls. Although the results on the Y-maze with the AD1 and AD2 are different, it is important to note that in the study which used larger groups of animals (i.e., AD1) there was a significant effect on this measure of short-term memory.

### Inoculation of *App*^*NL−F/NL−F*^ mice with AD-*S extracts* leads to greater amyloid accumulation in cortex and hippocampus

**Fig. 3:**
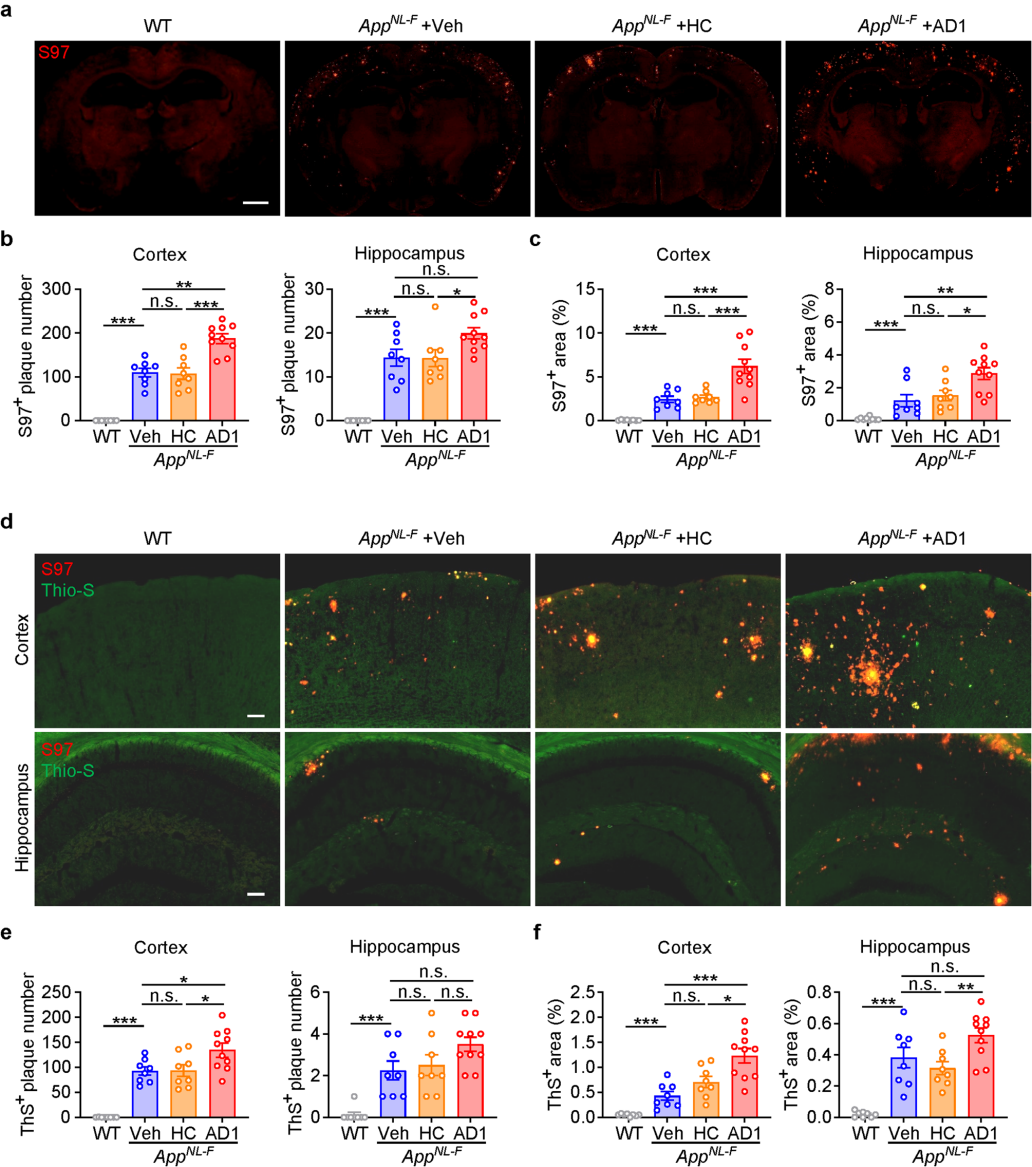
Inoculation of *App*^*NL−F/NL−F*^ mice with AD1 *S extract* accelerates amyloid accumulation in cortex and hippocampus. (**a**) Representative coronal brain sections of mice sacrificed immediately after behavior tests were completed (316–320 dpi). Global amyloid burden is visualized using the pan anti-Aβ polyclonal antibody S97 (red). Scale bar: 1 mm. (**b**) Quantification of S97-positive amyloid plaque numbers in the cortex and hippocampus: WT (*n* = 8), Veh (*n* = 8), HC (*n* = 8) and AD1 (*n* = 10). Cortex: Veh vs. WT, *p* < 0.001, *t* test. Cortex: AD1 vs. Veh, *p* = 0.0001; AD1 vs. HC, *p* < 0.001; F = 18.34, one-way ANOVA. Hippocampus: Veh vs. WT, *p* < 0.001, *t* test. Hippocampus: AD1 vs. Veh, *p* = 0.0466, Dunn’s multiple comparisons. (**c**) Quantification of the percentage of S97-positive plaque area in the cortex and hippocampus: WT (*n* = 8), Veh (*n* = 8), HC (*n* = 8) and AD1 (*n* = 10). Cortex: Veh vs. WT, *p* < 0.001, *t* test. Cortex: AD1 vs. Veh, *p* = 0.0003; AD1 vs. HC, *p* = 0.0006; F = 14.16, one-way ANOVA. Hippocampus: Veh vs. WT, *p* = 0.0002, Mann-Whitney tests. Hippocampus: AD1 vs. Veh, *p* = 0.0021, Kruskal–Wallis tests; AD1 vs. HC, *p* = 0.0270, Kruskal–Wallis tests. (**d**) Representative images showing the co-staining of S97 (red) and Thioflavin-S (ThS, green) in the cortex and hippocampus. Scale bar: 100 μm. (**e**) Quantification of ThS-positive plaque numbers in the cortex and hippocampus: WT (*n* = 8), Veh (*n* = 8), HC (*n* = 8) and AD1 (*n* = 10). Cortex: Veh vs. WT, *p* < 0.001, *t* test. Cortex: AD1 vs. Veh, *p* = 0.0354; AD1 vs. HC, *p* = 0.0404; F = 4.812, one-way ANOVA. Hippocampus: Veh vs. WT, *p* = 0.0005, *t* test. (**f**) Quantification of the percentage of ThS-positive plaque area in the cortex and hippocampus: WT (*n* = 8), Veh (*n* = 8), HC (*n* = 8) and AD1 (*n* = 10). Cortex: Veh vs. WT, *p* = 0.0006, *t* test. Cortex: AD1 vs. Veh, *p* = 0.0003; AD1 vs. HC, *p* = 0.0135; F = 11.73, one-way ANOVA. Hippocampus: Veh vs. WT: *p* = 0.0006, *t* test. Hippocampus: AD1 vs. HC: *p* = 0.0203, F = 4.542, one-way ANOVA. Significant differences are denoted as *p < 0.05, **p < 0.01, and ***P < 0.001. n.s. denotes not significant. Values are shown as mean ± SEM

Previous studies have shown that *App*^*NLF−/NL−F*^ mice begin to accumulate amyloid deposits in the cortex and hippocampus at around six months old, but significant staining is not seen until around fourteen months [24, 36]. Here, we collected brains of inoculated mice immediately upon completion of behavioral testing (from 316 to 320 dpi, i.e. when mice were ~ 12.5 months old). Consistent with prior longitudinal studies of *App*^*NLF−/NL−F*^ mice, immunostaining with a pan anti-Aβ polyclonal, S97, revealed limited staining in the cerebral cortex and hippocampus of the vehicle and HC-*S extract* inoculated mice. Strikingly, approximately twice as more S97 immunoreactivity was evident in brains of mice inoculated with AD1 and AD2 *S extracts* (Fig. 3a and Supplementary Fig. 3a). Notably, the numbers of S97^+^ plaques were significantly higher in the cortex of AD1 and AD2 inoculated mice versus vehicle and HC injected mice (Fig. 3b and Supplementary Fig. 3b). When quantifying the number of S97^+^ plaques in the hippocampus, a statistically significant difference was observed between AD1 and HC and between AD2 and vehicle. However, the comparison of AD1 versus vehicle did not reach statistical significance (Fig. 3b and Supplementary Fig. 3b). The percentage of brain area covered by S97^+^ staining showed a similar trend, and was significantly increased in the AD1 and AD2 groups versus the corresponding vehicle and HC groups (Fig. 3c and Supplementary Fig. 3c).

S97 recognizes multiple forms of Aβ (including Aβ in plaque and diffuse deposits) and was used to estimate total amyloid load. In contrast, ThS specifically recognizes plaque Aβ, but not diffuse deposits. Therefore, we conducted double-staining experiments with ThS to label dense plaques and S97 to label total Aβ (Fig. 3d and Supplementary Fig. 3d-e). Consistent with the expectation that Aβ is present in both plaques and diffuse deposits, the number of S97^+^ puncta was greater than the number of ThS^+^ plaques (Fig. 3e-f and Supplementary Fig. 3f-g). Importantly, as with S97^+^ puncta, mice inoculated with either AD1 or AD2 had significantly more cortical ThS^+^ plaques than vehicle or HC (Fig. 3e-f and Supplementary Fig. 3f-g). A similar trend was seen in the hippocampus, but the difference between AD *S extract* inoculated and vehicle inoculated mice did not reach statistical significance (Fig. 3e-f and Supplementary Fig. 3f-g). Nonetheless, it is evident that intracerebral inoculation of *App*^*NL−F/NL−F*^ mice with AD-*S extracts* accelerated global Aβ deposition.

### Inoculation of *App*^*NL−F/NL−F*^ mice with AD-*S extracts* induces microgliosis and astrocytosis

In *App*^*NL−F/NL−F*^ mice, activated microglia and astrocytes becomes evident when amyloid plaque formation starts, i.e. at around 6 months and activation increases with age and continued amyloid accumulation [24, 36]. Since significant acceleration of Aβ deposition was observed in mice at 316–320 dpi with AD *S extracts*, we asked whether treatment also induced gliosis. Microglia activation was evaluated through Iba1 staining and subsequently characterized by intensity, percentage of area, and morphology. Astrocytosis was visualized by GFAP immunostaining and subsequently quantitated by relative intensity and percentage of area.

**Fig. 4:**
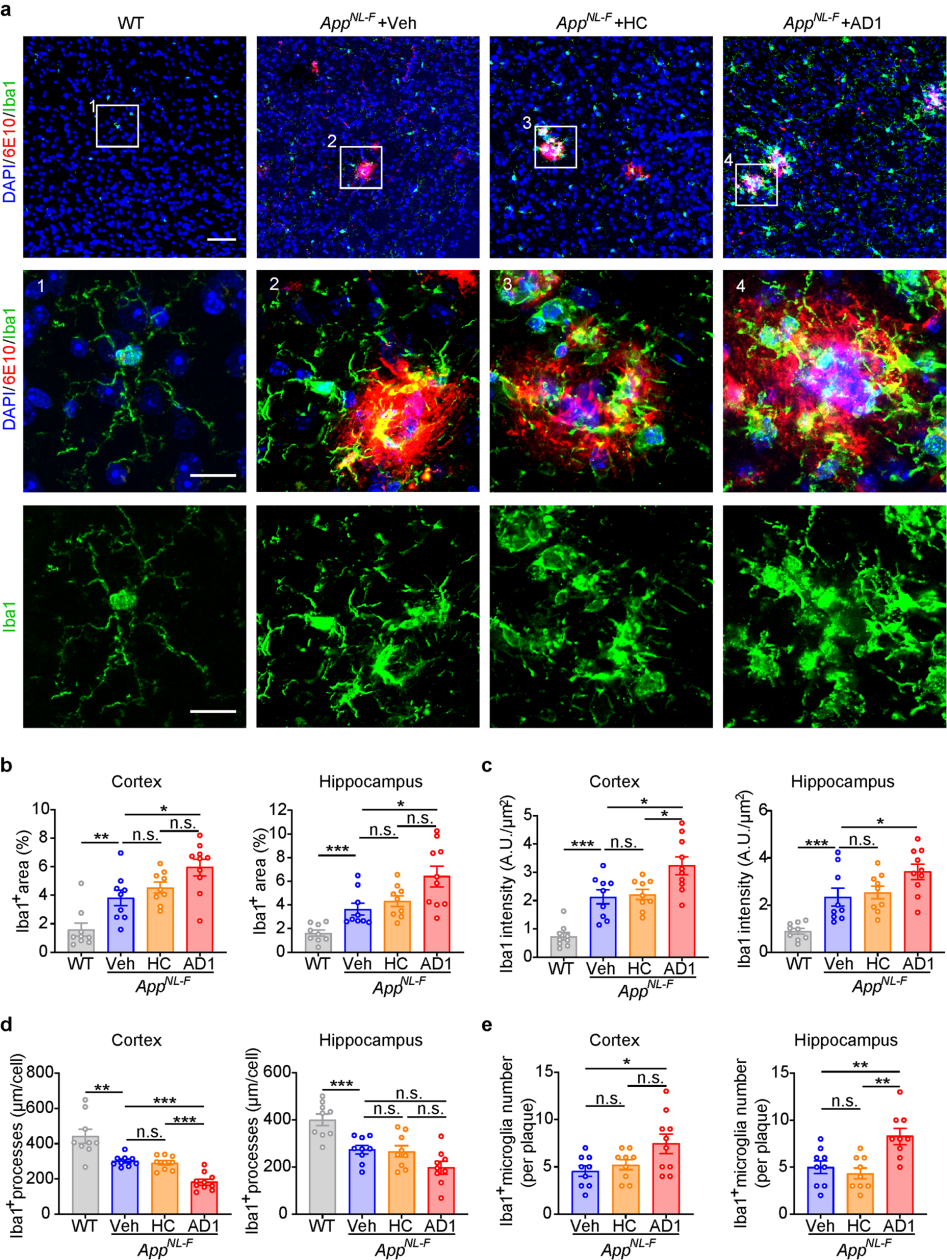
Inoculation of *App*^*NL−F/NL−F*^ mice with AD1 *S extract* induces microgliosis. (**a**) Representative images showing immunofluorescence staining of Iba1-positive microglia (green) and Aβ (6E10, red), together with DAPI (blue) for cell nuclei in the cortex. Boxed regions in upper panels (scale bar: 40 μm) are numbered and shown with a higher magnification in lower panels (scale bar: 10 μm) and illustrate different morphologies and activation states of microglia. (**b**) Quantification of the percentage of Iba1-positive microglia area in cortex and hippocampus: WT (*n* = 9), Veh (*n* = 9), HC (*n* = 9) and AD1 (*n* = 10). Cortex: Veh vs. WT, *p* = 0.0056, Mann-Whitney tests. Cortex: AD1 vs. Veh, *p* = 0.0139, F = 4.917, one-way ANOVA. Hippocampus: Veh vs. WT, *p* = 0.0002, Mann-Whitney tests. Hippocampus: AD1 vs. Veh, *p* = 0.0236, Kruskal–Wallis tests. (**c**) Quantification of Iba1 intensity relative to selected area in cortex and hippocampus: WT (*n* = 9), Veh (*n* = 9), HC (*n* = 9) and AD1 (*n* = 10). Cortex: Veh vs. WT, *p* = 0.0001, *t* test. Cortex: AD1 vs. Veh, *p* = 0.0109; AD1 vs. HC: *p* = 0.0192; F = 6.314, one-way ANOVA. Hippocampus: Veh vs. WT: *p* < 0.001, *t* test. Hippocampus: AD1 vs. Veh, *p* = 0.0435, Mann-Whitney tests. (**d**) Quantification of microglia processes by measuring the total length of branches per cell. Cortex: WT (*n* = 9), Veh (*n* = 9), HC (*n* = 9) and AD1 (*n* = 10). Cortex: Veh vs. WT, *p* = 0.0037, *t* test. Cortex: AD1 vs. Veh, *p* < 0.001; AD1 vs. HC, *p* < 0.001; F = 24.8, one-way ANOVA. Hippocampus: Veh vs. WT, *p* = 0.0008, *t* test. (**e**) The average number of Iba1-positive microglia associated with each plaque. Cortex: Veh (*n* = 9), HC (*n* = 9) and AD1 (*n* = 10). Hippocampus: Veh (*n* = 9), HC (*n* = 9) and AD1 (*n* = 9). Cortex: AD1 vs. Veh, *p* = 0.0226, F = 4.537, one-way ANOVA. Hippocampus: AD1 vs. Veh, *p* = 0.0060; AD1 vs. HC: *p* = 0.0011; F = 9.725, one-way ANOVA. Significant differences are denoted as *p < 0.05, **p < 0.01 and ***p < 0.001. n.s. denotes not significant. Values are shown as mean ± SEM

In brain sections of WT mice, we observed branched processes of microglia emanating from cell bodies with clear secondary and tertiary branching consistent with “radial microglia” in a homeostatic state (Fig. 4a). In contrast, *App*^*NL−F/NL−F*^ mice treated with vehicle or HC at 316–320 dpi displayed signs of microglia activation, with microglia surrounding dense amyloid plaques, having fewer and shorter processes. Moreover, Iba1 intensity was higher in brains of *App*^*NL−F/NL−F*^ mice treated with vehicle or HC to WT (Fig. 4a). Consistent with their higher amyloid load, *App*^*NL−F/NL−F*^ mice inoculated with AD1 or AD2 had more extensive activation of Iba1^+^ microglia across the whole cortex (Fig. 4a and Supplementary Fig. 4a). The percentage of area covered Iba1^+^ microglia was significantly higher in AD1 and AD2 inoculated mice than in vehicle inoculated mice, while no significant difference was seen between HC inoculated and vehicle inoculated mice (Fig. 4b and Supplementary Fig. 4b). The average Iba1 intensity in the AD1 and AD2 groups, both in cortex or hippocampus, was significantly higher than that in the vehicle or HC group (Fig. 4c and Supplementary Fig. 4c). Moreover, we also found that the complexity of processes emanating from microglia was significantly reduced in the cortex and hippocampus of *App*^*NL−F/NL−F*^ mice compared to age-matched WT mice. Treatment with AD-*S extracts* further decreased microglial processes complexity in the cortex but not hippocampus, relative to vehicle and HC inoculated mice (Fig. 4d and Supplementary Fig. 4d). The number of Iba1^+^ microglia associated with plaques was also significantly higher in the hippocampus of AD1-inoculated mice, despite the fact that there was only a trend detected in the cortex. Interestingly, both brain regions of AD2-inoculated mice had significantly more activated microglia associated with plaques than vehicle or HC inoculated mice (Fig. 4e and Supplementary Fig. 4e).

**Fig. 5:**
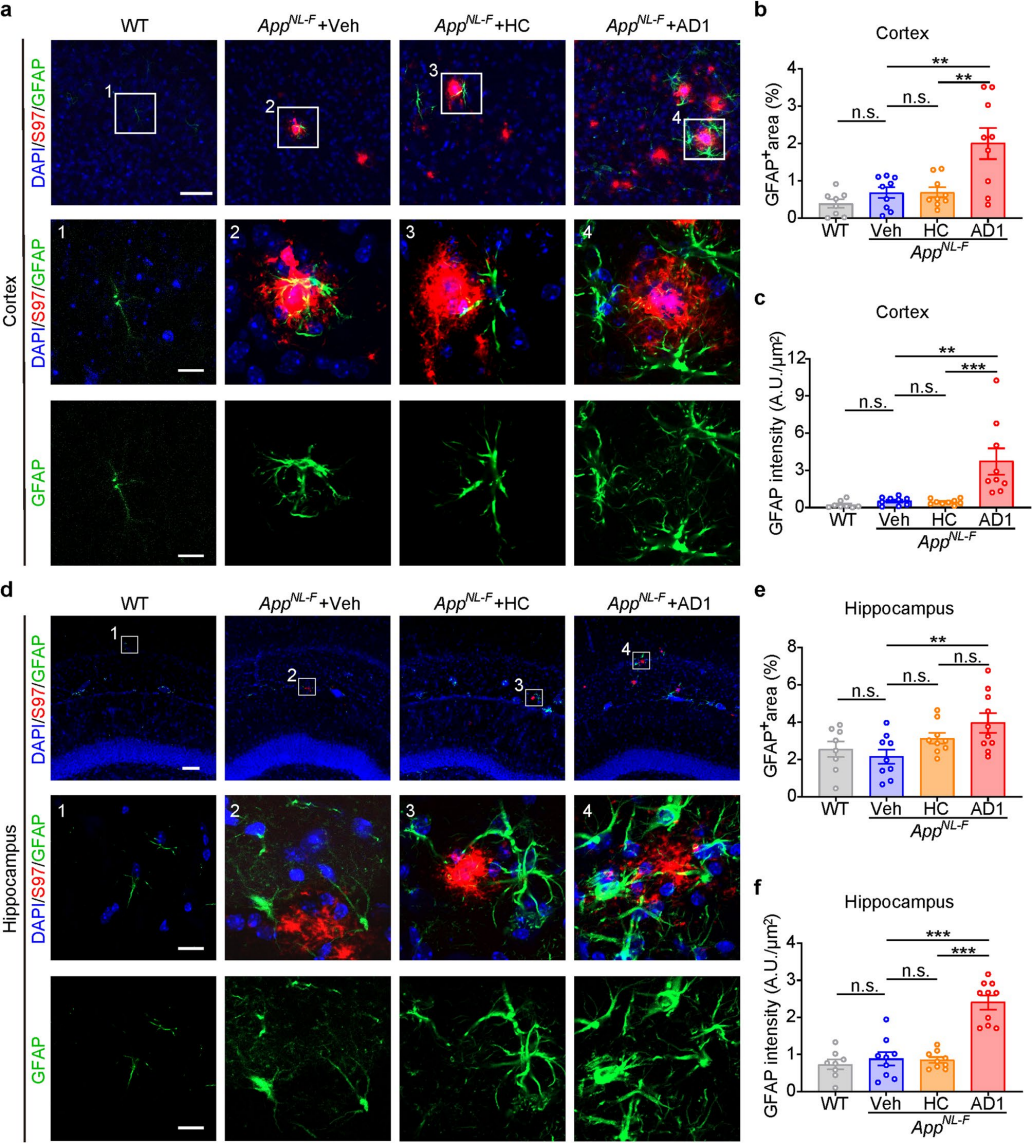
Inoculation of *App*^*NL−F/NL−F*^ mice with AD1 *S extract* induces astrocytosis. (**a**,** d**) Representative images showing immunofluorescence staining of GFAP-positive astrocyte (green) and Aβ (S97, red), together with DAPI (blue) for cell nuclei in the cortex and hippocampus. Boxed regions in upper panels (scale bar: 40 μm) are numbered and shown with a higher magnification in lower panels (scale bar: 10 μm) and illustrate the astrocytosis associated with amyloid plaques. (**b**) Quantification of the percentage of GFAP-positive astrocyte area in cortex: WT (*n* = 8), Veh (*n* = 9), HC (*n* = 9) and AD1 (*n* = 9). AD1 vs. Veh, *p* = 0.0035; AD1 vs. HC, *p* = 0.0038; F = 8.788, one-way ANOVA. (**c**) Quantification of GFAP intensity relative to selected area in cortex: WT (*n* = 8), Veh (*n* = 9), HC (*n* = 9) and AD1 (*n* = 9). AD1 vs. Veh, *p* = 0.0015; AD1 vs. HC, *p* = 0.0005; Kruskal–Wallis tests. (**e**) Quantification of the percentage of GFAP-positive astrocyte area in hippocampus: WT (*n* = 8), Veh (*n* = 9), HC (*n* = 9) and AD1 (*n* = 10). AD1 vs. Veh, *p* = 0.0098, F = 5.151, one-way ANOVA. (**f**) Quantification of GFAP intensity relative to selected area in hippocampus: WT (*n* = 8), Veh (*n* = 9), HC (*n* = 9) and AD1 (*n* = 10). AD1 vs. Veh, *p* < 0.001; AD1 vs. HC, *p* < 0.001; F = 34.33, one-way ANOVA. Significant differences are denoted as *p < 0.05, **p < 0.01, and ***p < 0.001. n.s. denotes not significant. Values are shown as mean ± SEM

Minimal GFAP^+^ staining was observed surrounding plaques in the cortex and hippocampus of *App*^*NL−F/NL−F*^ mice treated with vehicle or HC *S extract* (Fig. 5a and Supplementary Fig. 5a). However, a dramatic increase of GFAP intensity and percentage of GFAP^+^ area was observed in the cortex of mice inoculated with AD1 *S extracts*, when compared to vehicle or HC extract (Fig. 5b and **c**). In the hippocampus, GFAP intensity and GFAP^+^ area of AD1 inoculated mice were significantly higher than the vehicle group (Fig. 5e and **f**). Compared to the HC group, the AD1 group showed significantly higher GFAP intensity, but only a trend toward an increase of the GFAP^+^ area (Fig. 5e and **f**). Moreover, GFAP^+^ area and intensity in the cortex and hippocampus of AD2 inoculated mice were significantly higher than vehicle (Supplementary Fig. 5c-f). These data provide convincing data that intracerebral injecting of AD *S extracts* in *App*^*NL−F/NL−F*^ mice accelerates plaque-associated pathology such as astrocytosis.

### Inoculation of *App*^*NL−F/NL−F*^ mice with bioactive AD *S extracts* induces neuronal dystrophy and synaptic loss

The neurodegenerative process in AD is characterized by extensive synaptic and neuritic damage and subsequent neuronal loss, with synaptic loss the best neuropathological correlate of cognitive decline in AD [38, 39]. Prior studies and our current work have shown that soluble extracts of human AD cortical tissue caused significant impairment of neurite integrity and synaptic plasticity in multiple in vivo and in vitro paradigms [19, 34]. Given the potent bioactivity and ability to accelerate amyloid deposits, we asked whether the memory impairment observed in AD1 and AD2 inoculated mice was linked to neuronal dystrophy and/or synaptic loss.

**Fig. 6:**
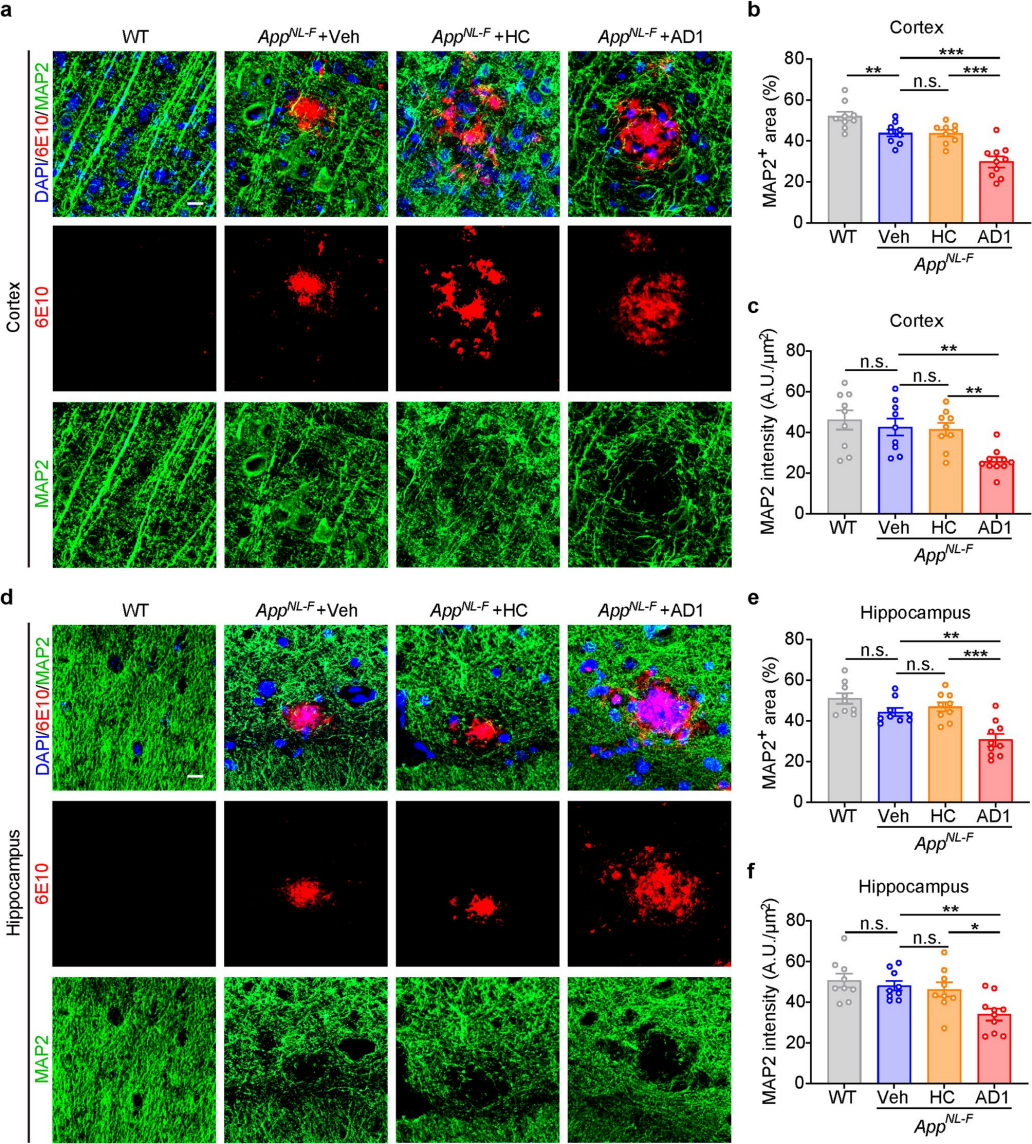
Inoculation of *App*^*NL−F/NL−F*^ mice with AD1 *S extract* induces neuronal dystrophy. (**a**,** d**) Representative images showing immunofluorescence staining of dendritic marker MAP2 (green) and Aβ (6E10, red), together with DAPI (blue) for cell nuclei in the cortex and hippocampus. Scale bar: 10 μm. (**b**) Quantification of the percentage of MAP2-positive area in cortex: WT (*n* = 9), Veh (*n* = 9), HC (*n* = 9) and AD1 (*n* = 10). Veh vs. WT, *p* = 0.0093, *t* test. AD1 vs. Veh, *p* = 0.0002; AD1 vs. HC, *p* = 0.0002; F = 15.81, one-way ANOVA. (**c**) Quantification of MAP2 intensity relative to selected area in cortex: WT (*n* = 9), Veh (*n* = 9), HC (*n* = 9) and AD1 (*n* = 10). AD1 vs. Veh, *p* = 0.0025; AD1 vs. HC, *p* = 0.0047; F = 9.027, one-way ANOVA. (**e**) Quantification of the percentage of MAP2-positive area in hippocampus: WT (*n* = 9), Veh (*n* = 9), HC (*n* = 9) and AD1 (*n* = 10). AD1 vs. Veh, *p* = 0.0012; AD1 vs. HC, *p* = 0.0002; F = 13.57, one-way ANOVA. (**f**) Quantification of MAP2 intensity relative to selected area in hippocampus: WT (*n* = 9), Veh (*n* = 9), HC (*n* = 9) and AD1 (*n* = 10). AD1 vs. Veh, *p* = 0.0060; AD1 vs. HC, *p* = 0.0176; F = 6.995, one-way ANOVA. Significant differences are denoted as *p < 0.05, **p < 0.01 and ***p < 0.001. n.s. denotes not significant. Values are shown as mean ± SEM

MAP2 immunostaining revealed dystrophic and fragmented dendrites surrounding plaques in the cortex of *App*^*NL−F/NL−F*^ mice, whereas in age-matched WT mice dendrites were healthy with a thick filament-like appearance (Fig. 6a). At 316–320 dpi, the fragmentation of neurites and loss of structural network in cortex became more severe in mice that received AD1 and AD2 brain extracts compared to mice inoculated with HC or vehicle (Fig. 6a and Supplementary Fig. 6a). The MAP2^+^ dendrites in the hippocampus were more disrupted in AD1 and AD2 inoculated mice, than in HC or vehicle treated mice (Fig. 6d and Supplementary Fig. 6b). Statistical analysis confirmed the difference in MAP2^+^ area and intensity between mice inoculated with AD1 *S extract* versus mice inoculated with HC *S extract* or vehicle (Fig. 6b-c, and **e-f**). The cortical MAP2^+^ area of AD2 inoculated mice was significantly lower than vehicle inoculated mice (Supplementary Fig. 6c), whereas the MAP2 intensity between them was not statistically different (Supplementary Fig. 6d). Although the MAP2^+^ area and intensity in the hippocampus of AD2 inoculated tended to be lower than vehicle group, they did not reach statistical significance (Supplementary Fig. 6c-f).

**Fig. 7:**
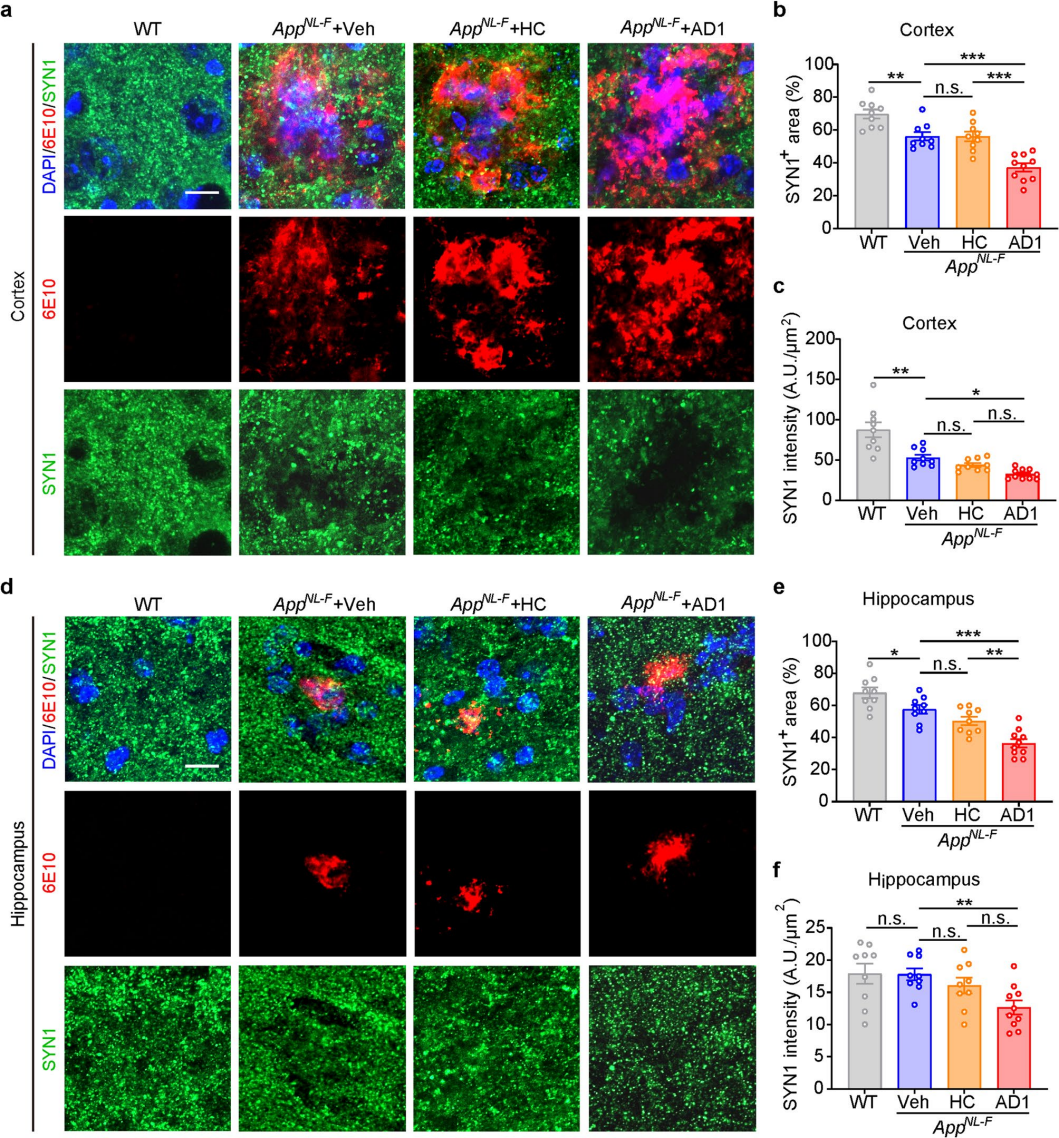
Inoculation of *App*^*NL−F/NL−F*^ mice with AD1 *S extract* induces synaptic loss. (**a**,** d**) Representative images showing immunofluorescence staining of synaptic marker SYN1 (green) and Aβ (6E10, red), together with DAPI (blue) for cell nuclei in the cortex and hippocampus. Scale bar: 10 μm. (**b**) Quantification of the percentage of SYN1-positive area in cortex: WT (*n* = 9), Veh (*n* = 9), HC (*n* = 9) and AD1 (*n* = 10). Veh vs. WT, *p* = 0.0022, *t* test. AD1 vs. Veh, *p* = 0.0001; AD1 vs. HC, *p* < 0.001; F = 16.89, one-way ANOVA. (**c**) Quantification of SYN1 intensity relative to selected area in cortex: WT (*n* = 9), Veh (*n* = 9), HC (*n* = 9) and AD1 (*n* = 10). Veh vs. WT, *p* = 0.0031, *t* test. AD1 vs. Veh, *p* = 0.0337, F = 21.85, one-way ANOVA. (**e**) Quantification of the percentage of SYN1-positive area in hippocampus: WT (*n* = 9), Veh (*n* = 9), HC (*n* = 9) and AD1 (*n* = 10). Veh vs. WT, *p* = 0.0208, *t* test. AD1 vs. Veh, *p* < 0.001; AD1 vs. HC, *p* = 0.0026; F = 16.96, one-way ANOVA. (**f**) Quantification of SYN1 intensity relative to selected area in hippocampus: WT (*n* = 9), Veh (*n* = 9), HC (*n* = 9) and AD1 (*n* = 10). AD1 vs. Veh, *p* = 0.0058, F = 6.140, one-way ANOVA. Significant differences are denoted as *p < 0.05, **p < 0.01, and ***p < 0.001. n.s. denotes not significant. Values are shown as mean ± SEM

We also performed immunostaining for synapsin-1 (SYN1), the most abundant synaptic protein, to examine whether inoculation with AD *S extracts* had effected synapses (Fig. 7 and Supplementary Fig. 7). In the cortex of *App*^*NL−F/NL−F*^ mice, reduced SYN1 immunoreactivity was observed around amyloid plaques, whereas this reduction was less pronounced in the hippocampus (Fig. 7a and **d**). At 316–320 dpi, the reduction of SYN1^+^ staining became more severe in the mice inoculated with AD1 and AD2 extracts than mice treated with vehicle or HC (Fig. 7a and **b**, Supplementary Fig. 7a and **b**). Statistical analysis confirmed the difference in SYN1^+^ area in cortex and hippocampus between mice inoculated with AD1 and AD2 versus mice inoculated with vehicle or HC (Fig. 7b and **e**, Supplementary Fig. 7c and **e**). Moreover, SYN1 intensity in the cortex and hippocampus was significantly lower in the brains of mice treated with AD1 compared to the vehicle group, but not statistically different from the HC group (Fig. 7c and **f**). Similarly, SYN1 intensity in the cortex of AD2-inoculated mice was significantly lower than vehicle-inoculated mice (Supplementary Fig. 7d). A similar trend was seen in the hippocampus, but the difference between AD2 and vehicle groups did not reach statistical significance (Supplementary Fig. 7f).

## Discussion

Despite significant advances in our understanding of AD, the precise forms of Aβ which contribute to disease and the mechanisms by which they act remain obscure. Nonetheless, it is clear that antibodies which facilitate removal of amyloid have therapeutic benefit, and that the benefit is directly linked to extent of amyloid lowering [40]. While removal of amyloid is now an established means to treat disease, it seems reasonable that disease could be prevented by avoiding the accumulation of amyloid. Thus, there is great interest in preventing the formation and proliferation of cerebral Aβ deposits. One avenue to achieve this end is to target the seeds which propagate amyloid pathology.

Prior studies of Aβ seeding have relied on synthetic aggregates or crude homogenates of human or animal brains. In vivo seeding using aggregates of synthetic Aβ typically required high concentrations and multiple injections, and only modestly accelerated amyloid deposition with minimal effects on behaviors [41–45]. In contrast, biological seeds, especially from postmortem human brain tissues, have long-lasting seeding activity that persist for decades and require only a single inoculation [10, 11]. Indeed, a recent study showed that apparently very low levels of cerebral Aβ (30 µL of a 0.001% (w/v) crude AD brain homogenate) was sufficient to seed amyloid formation in *App*^*NL−F/NL−F*^ mice [46]. However, the molecular characteristics of seeds have not been defined.

Recently we developed a minimally disruptive method to extract bioactive Aβ from frozen postmortem brain and found that the Aβ in these so-called *S extracts* represent only a minority of the total aqueously extractable Aβ [19]. While these *S extracts* contain less Aβ and have higher toxic activity, than traditional homogenates they contain a mixture of Aβ species. These include high molecular weight forms of Aβ detected by SEC [18, 19] and protofibrils visualized by electron microscopy [34] plus a variety of intermediate and low molecular weight Aβ species detected by SEC [18, 19]. As yet it is not known whether all or just some of these different species contribute to bioactivity and if any of them are capable of seeding Aβ aggregation in vivo.

To address issue of seeding, we inoculated *App*^*NL−F/NL−F*^ mice with well-characterized *S extracts* from AD brain, and assessed their effects on: (i) amyloid accumulation and associated pathologies, and (ii) behavioral paradigms of working and spatial reference memory. For these experiments the principal biological comparisons were between *App*^*NL−F/NL−F*^ mice inoculated with AD *S extract* versus *App*^*NL−F/NL−F*^ mice inoculated with control *S extract* and vehicle. WT C57BL/6J which are not a perfect match for *App*^*NL−F/NL−F*^ provided a positive control for behavioral studies and a negative control for immunohistological analyses.

We found that a single inoculation of AD-*S extracts* at 8 weeks old was sufficient to enhance Aβ deposition in *App*^*NL−F/NL−F*^ mice when tested at 316–320 dpi. This observation demonstrates that certain form(s) of diffusible Aβ in AD brain potently catalyze the deposition of amyloid in vivo. While *S extracts* contain a mixture of Aβ species this preparation is considerably more refined than AD brain-derived inocula tested heretofore and provide a source from which yet better-defined species can be isolated and tested. In future studies we intend fractionating *S extracts* (such as those from AD1 and AD2) and testing their acute bioactivity and ability to seed aggregation in *App*^*NL−F/NL−F*^ mice. Thereafter, the Aβ content of the most potent fractions should be analyzed. Furthermore, by comparing seeding activity versus in vitro readouts such neuritotoxicity it should shed light on the relationship between acute toxicity and seeding activity, and/or effects on neuronal integrity.

*App*^*NL−F/NL−F*^ mice model the very earliest stages of AD, exhibiting mild functional deficits without tau pathology, and neurodegeneration only observable at very advanced ages [24]. In keeping with prior studies, vehicle injected *App*^*NL−F/NL−F*^ mice at 300 dpi (approximate 12 months old) behaved normally on the behavioral tasks examined. In contrast, AD1-inoculated mice exhibited significant impairments in both the Barnes-maze and Y-maze. AD2 inoculated mice showed similar impaired recall on the Barnes mazes, but did not evince significant effects in Y-maze performance. The divergence in performance on the Y-maze between AD1 and AD2 is likely to be a consequence of the smaller number of AD2 inoculated mice tested in the Y-maze. Nonetheless, in future studies it will be important to test the effects of *S extracts* from other brains to determine if differences in Aβ content effect learned behavior.

In addition to age-dependent accumulation of amyloid plaques and cognitive decline, *App*^*NL−F/NL−F*^ mice also develop other AD relevant pathologies, including synaptic loss, and microgliosis [47]. Activation of microglia was readily evident in approximate 12.5-month old *App*^*NL−F/NL−F*^ mice with the percentage of Iba1^+^ area, Iba1 intensity and processes, significantly affected compared with age-matched WT mice (Fig. 4). However, we did not observe significant astrocytosis in the same mice, indicating asynchronous responses of these two types of glial cells (Fig. 5). Nonetheless, inoculation of AD1 and AD2 extracts resulted in higher levels of activation of both microglia and astrocytes associated with amyloid plaques. This finding is consistent with prior studies which indicate that gliosis is directly linked to overall cerebral Aβ load [48].

Given that *S extracts* from AD1 and AD2 had similarly increased amyloid load and associated gliosis, yet these extracts had different levels of in vitro neuritotoxic activity (Supplementary Fig. 1), it would appear that the neuritotoxins present in the initial inocula did not differential effect either the induction of amyloid deposition or the associated gliosis. However, the impairment to dendrites and synapses seen with AD1 was more severe than with AD2. While it is interesting to speculate that the neuronal and synaptic loss seen in inoculated mice may be related to the in vitro neuritotoxicity of *S extracts*, study of *S extracts* from many more AD brains with a range of in vitro neuritotoxic activities will be required. Additional studies should also incorporate analyses at different time points post-inoculation to better understand the relationship between the acceleration of amyloid deposition and the appearance of behavioral deficits. As such the current report provides a spring board for these future studies.

Tau pathology was not characterized in our study, as previous work showed that it cannot be induced by human Aβ seeds unless inoculated into AD transgenic mouse models with tau pathology [17, 49]. However, a few studies reported that tau pathology can be detected in Aβ plaque-bearing APPswe/PS1dE9 mice and *Microcebus murinus* that received inoculation of human AD brain extracts [6, 14]. In future it will be important to use mouse lines that bear human microtubule-associated protein tau (Mapt) gene and expresses all six human tau isoforms. Indeed, the recently generated human MAPT KI-*App*^*NL−F/NL−F*^ mouse line should be ideal model to study the Aβ-tau axis [50]. Moreover, tau derived from AD patients’ CSF can accelerate tau pathology in transgenic mice [51], while Aβ in the CSF of certain transgenic mice does not show seeding activity [52]. These studies suggest that biologically active tau seeds reach the CSF compartment in human but Aβ seeds in animals may not [53]. In future it will be important to search for Aβ seeds in human CSF and investigate the in vivo seeding properties.

## Conclusions

Here, we demonstrated that *S extracts* from human AD brains contain a range of Aβ assemblies and primary sequences, and that one or more of these components disrupt neurite integrity of iPSC-derived neurons. A single injection of such AD *S extracts* accelerated cerebral amyloid deposition which was accompanied by microgliosis, astrocytosis, neuronal dystrophy and synaptic loss. Importantly, mice tested at similar post-inoculation interval exhibited significant decline of working and learning memory of. Thus, our data demonstrate that diffusible Aβ derived from AD brain are highly toxic and can accelerate amyloid deposition. Further fractionation of the AD *S extracts* should allow elucidation of the components mediating these important pathogenic effects and in turn enable therapeutic targeting of these species.

## Electronic supplementary material

Below is the link to the electronic supplementary material.

Supplementary Material 1

## Data Availability

No datasets were generated or analysed during the current study.

## Abbreviations

Aβ: Amyloid β-protein
AD: Alzheimer’s disease
iPSC: Induced Pluripotent Stem Cells
WT: Wild-type
LTP: Long-term potentiation
HC: Healthy control
GuHCl: Guanidine hydrochloride
IACUC: Institutional Animal Care and Use Committees
PCR: Polymerase chain reaction
PMI: Postmortem interval
aCSF-B: Artificial cerebrospinal fluid base buffer
ID: Immunodepleted
MSD: Meso Scale Discovery
iNs: Induced neurons
dpi: Days post-inoculation
RP-HPLC: Reverse-Phase High-Performance Liquid Chromatography
SEC: Size exclusion chromatography
Ngn2: Neurogenin 2
GFP: Green Fluorescent Protein
rtTA: Reverse tetracycline transcriptional activator
D-PBS: Dulbecco’s Phosphate-Buffered Saline
PBS: Phosphate-Buffered Saline
PFA: Paraformaldehyde
OCT: Optimal cutting temperature
BSA: Bovine Serum Albumin
DAPI: 6-diamidino-2-phenylindole
ThS: Thioflavin-S
Iba1: Ionized Calcium Binding Adaptor Molecule 1
GFAP: Glial fibrillary acidic protein
SYN1: Synapsin I
MAP2: Microtubule Associated Protein 2
ANOVA: Analysis of variance
BD: Blue dextran
Mapt: Microtubule-associated protein tau
Veh: Vehicle
