# Supplementary material for: Experimental evidence that readily diffusible forms of Aβ from Alzheimer’s disease brain have seeding activity
Source: Acta Neuropathol Commun. 2025 May 24;13:112. doi: 10.1186/s40478-025-02032-w (PMC12102860; doi:10.1186/s40478-025-02032-w)
Supplement: Supplementary file 1 — Supplementary Material 1 [file 40478_2025_2032_MOESM1_ESM.pdf]

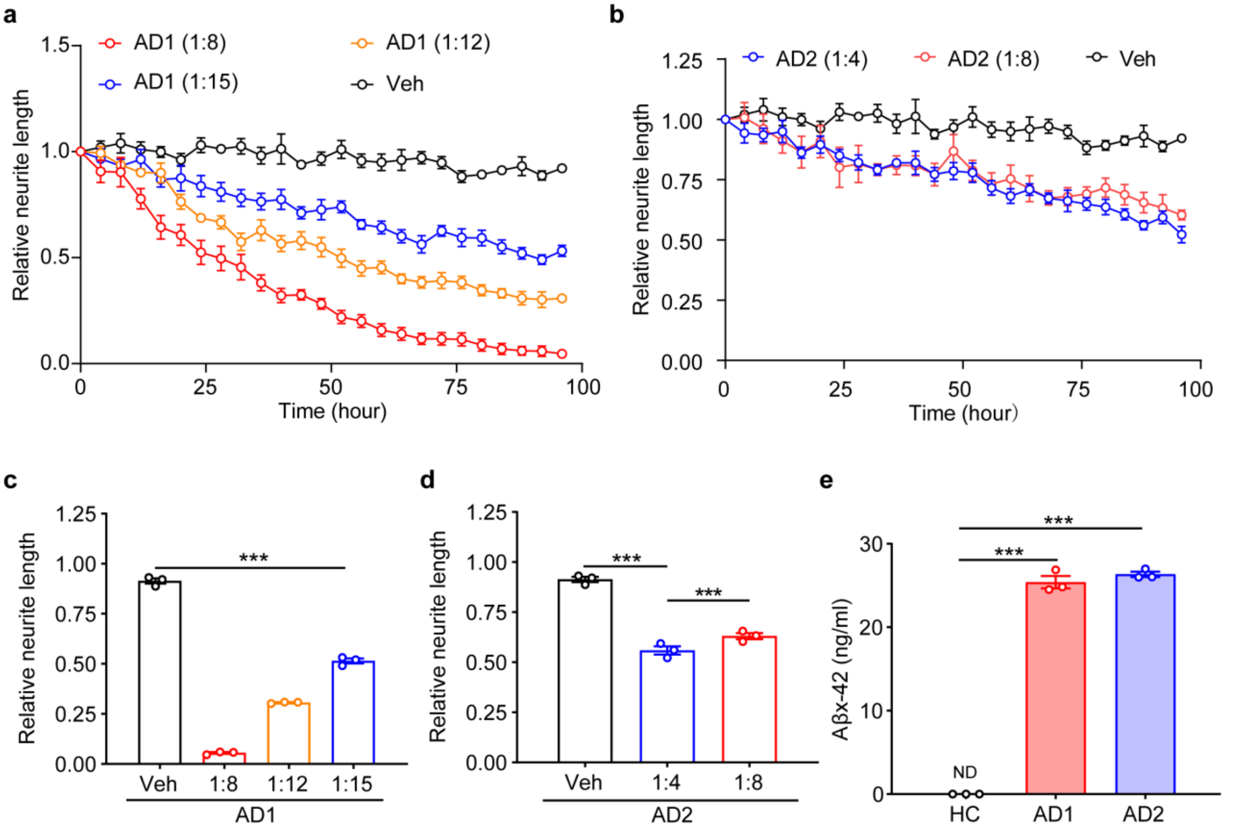

Supplementary Figure 1. *S extracts* of human AD brains cause a time- and dose-dependent loss of neuritic complexity. (**a, b**) Human iPSC-derived neurons (iNs) were treated with AD1 and AD2 brain extracts at different dilutions and cells imaged for 96 hours. NeuroTrack-identified neurite length was calculated relative to baseline collected 6 h prior to sample application. Values are the average of triplicate wells ± SEM. (**c, d**) Histogram plots of normalized neurite length are derived from the last 3 recordings the traces shown in **a** and **b**. Values are the average of triplicate wells ± SEM. (**e**) Aβx−42 immunoassay quantifies Aβ in HC, AD1 and AD2 brain extracts used for iNs treatment. Significant differences are denoted as ***p<0.001.

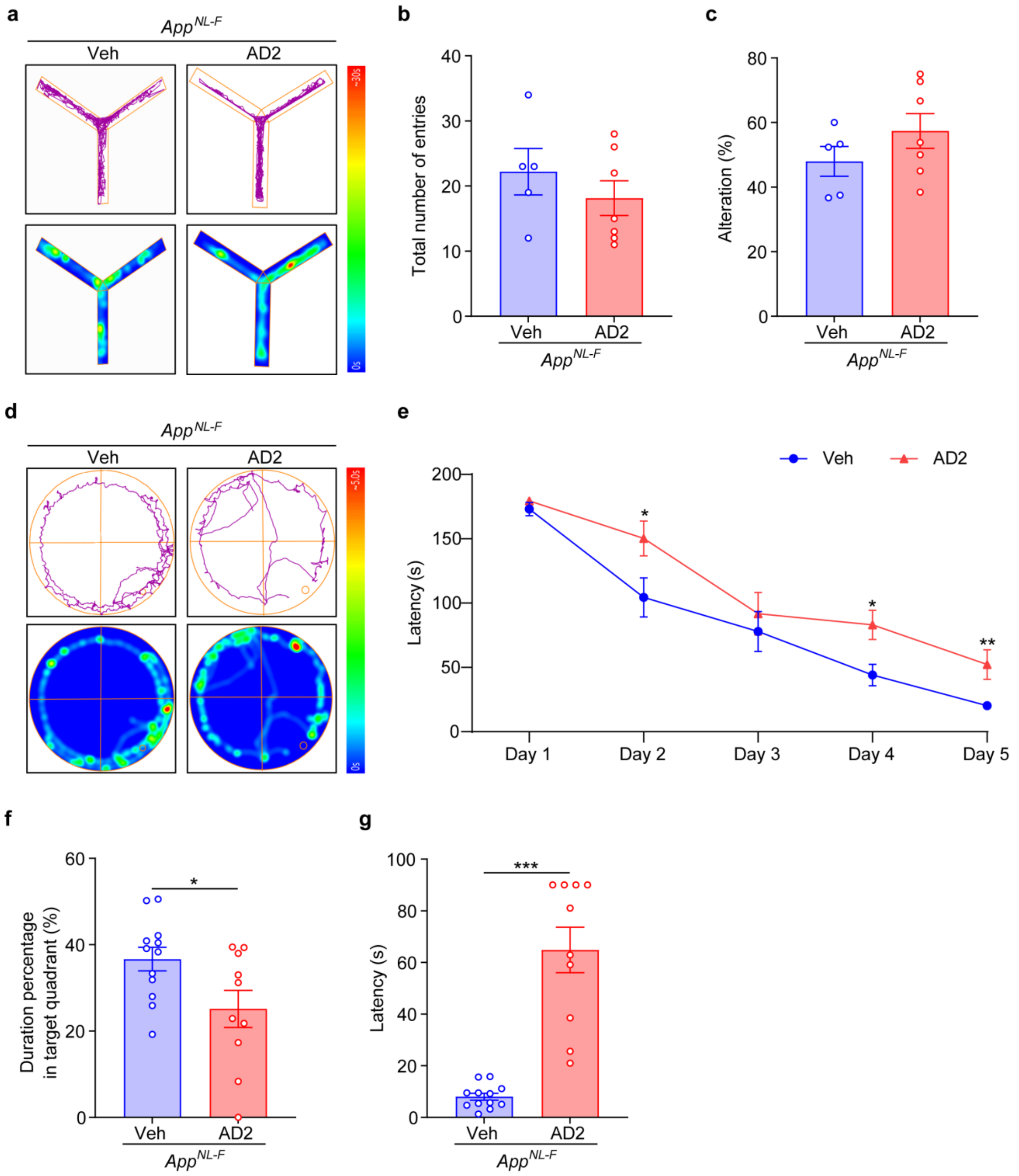

Supplementary Figure 2. Intracerebral inoculation of *App*$^{NL\text{-}F/NL\text{-}F}$ mice with AD2 *S extract* impairs spatial learning and memory. (**a**) Representative trajectories and heat maps of mice in Y-maze task. (**b, c**) Total number of mice entries the three arms and the percentage of correct alterations

in Y-maze: Veh (n=5), AD2 (n=7). (**d**) Representative trajectories and heat maps of mice in Barnes-maze. (**e**) The primary latency to find the escape tunnel across the acquisition phase for 5 days in Barnes-maze: Veh (n=12), AD2 (n=10). Day 2: AD2 vs Veh, p=0.0394; Day 4: AD2 vs Veh, p=0.0107; Day 5: AD2 vs Veh, p=0.0076, t test. (**f**) The percentage of time spent in the target quadrant during the probe test in Barnes-maze: Veh (n=12), AD2 (n=10). AD2 vs Veh, p=0.029, t test. (**g**) The primary latency to find the escape tunnel during the probe test in Barnes-maze: Veh (n=12), AD2 (n=10). AD2 vs Veh, p<0.001, Mann-Whitney test. Significant differences are denoted as *p<0.05, **p<0.01 and ***p<0.001. All values are shown as mean ± SEM.

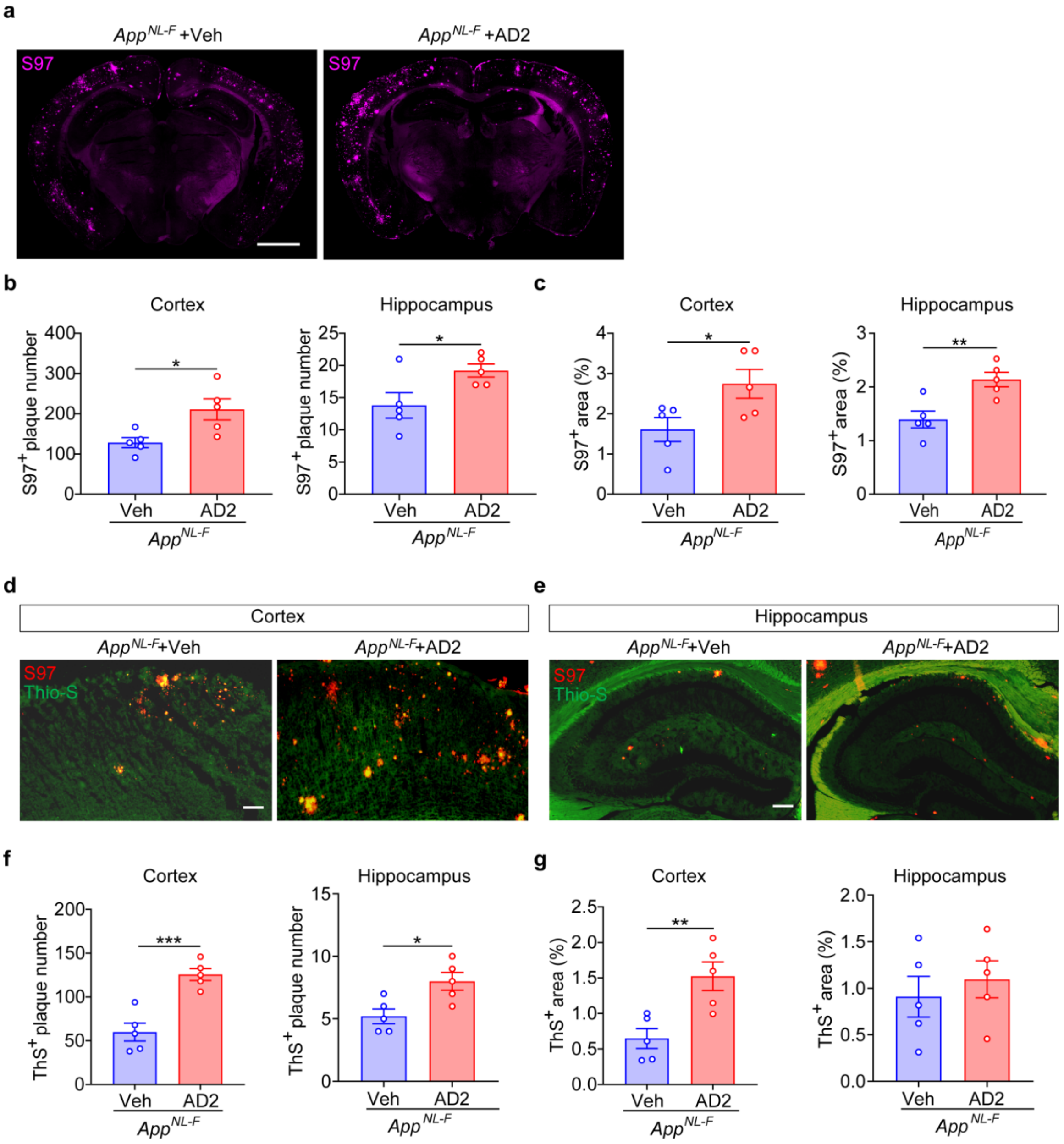

Supplementary Figure 3. Inoculation of *App*$^{NL\text{-}F/NL\text{-}F}$ mice with AD2 *S extract* accelerates amyloid accumulation in cortex and hippocampus. (**a**) Representative coronal brain sections of mice sacrificed immediately after behavior tests were completed. Global amyloid burden is visualized by a pan anti- Aβ polyclonal antibody S97 (purple). Scale bar: 1 mm. (**b**) Quantification of S97-positive amyloid plaque numbers in the cortex and hippocampus of *App*$^{NL\text{-}F/NL\text{-}F}$ mice inoculated

with vehicle (n=5) or AD2 *S extract* (n=5). Cortex: AD2 vs Veh, p=0.0213; Hippocampus: AD2 vs Veh, p=0.0419, t test. (**c**) Quantification of the percentage of S97-positive plaque area in the cortex and hippocampus of $App^{NL\text{-}F/NL\text{-}F}$ mice inoculated with vehicle (n=5) or AD2 brain extract (n=5). Cortex: AD2 vs Veh, p = 0.0411; Hippocampus: AD2 vs Veh, p=0.0070, unpaired t test. (**d, e**) Representative images showing the co-staining of S97 (red) and Thio-S (ThS, green) in the cortex and hippocampus. Scale bar: 100 µm. (**f**) Quantification of ThS-positive plaque numbers in the cortex and hippocampus of $App^{NL\text{-}F/NL\text{-}F}$ mice inoculated with vehicle (n=5) or AD2 *S extract* (n=5). Cortex: AD2 vs Veh, p=0.0007, t test; Hippocampus: AD2 vs Veh, p=0.0157, t test. (**g**) Quantification of the percentage of ThS-positive plaque area in the cortex and hippocampus of $App^{NL\text{-}F/NL\text{-}F}$ mice inoculated with vehicle (n=5) or AD2 *S extract* (n=5). Cortex: AD2 vs Veh, p=0.0071, t test; Hippocampus, AD2 vs Veh, p=0.5457, t test. Significant differences are denoted as *p<0.05, **p<0.01 and ***p<0.001. Values are shown as mean ± SEM.

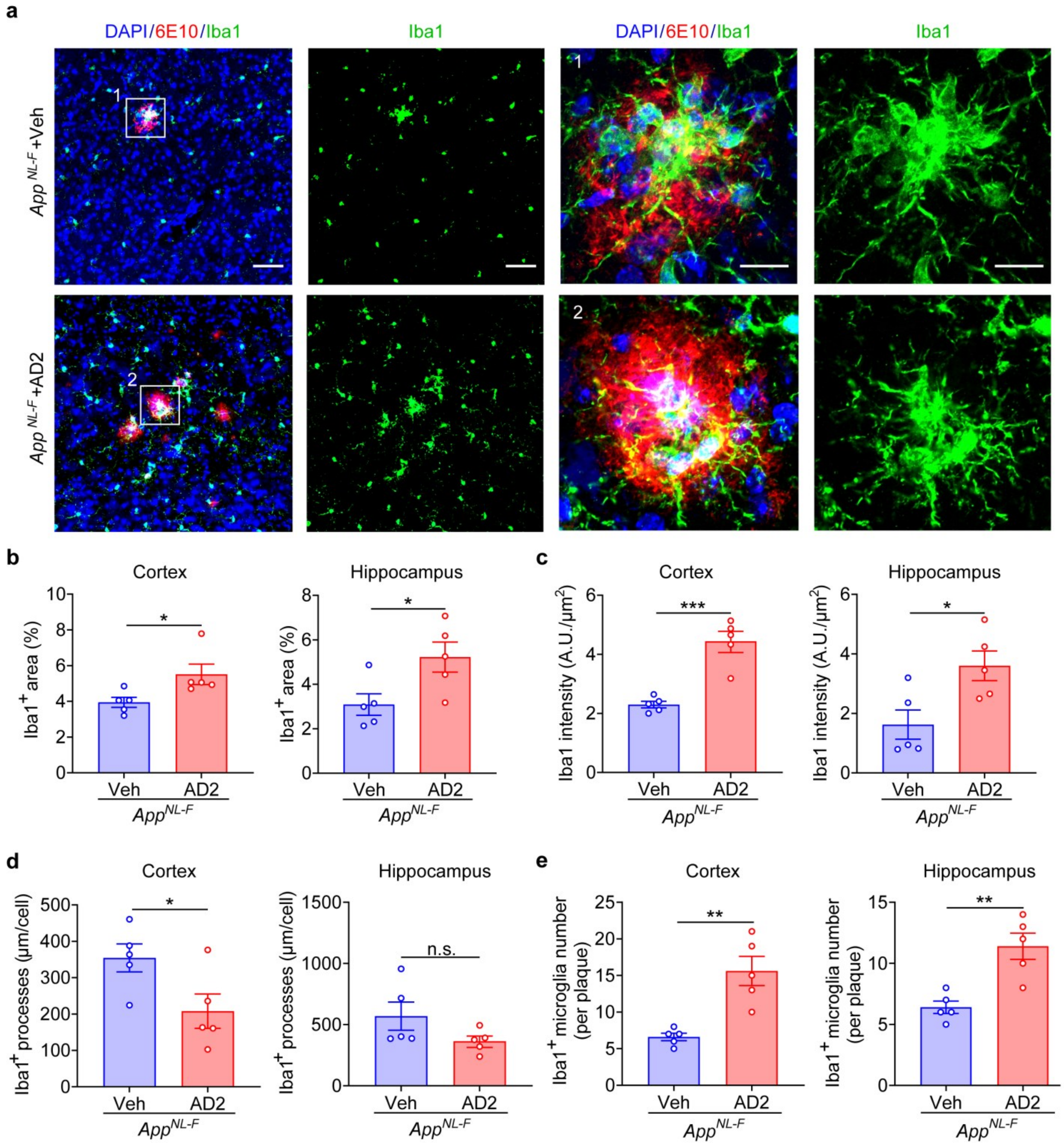

Supplementary Figure 4. Inoculation of *App$^{NL-F/NL-F}$* mice with AD2 *S extract* induces microgliosis.

(**a**) Representative images showing immunofluorescence staining of Iba1-positive microglia (green) and Aβ (6E10, red), together with DAPI (blue) for cell nuclei in the cortex. Boxed regions in left panels (scale bar: 40 μm) are numbered and shown with a higher magnification in right

panels (scale bar: 10 µm) and illustrate different morphologies and activation states of microglia. (**b**) Quantification of the percentage of Iba1-positive microglia area in the cortex and hippocampus of *App*$^{NL-F/NL-F}$ mice inoculated with vehicle (n=5) or AD2 brain extract (n=5). Cortex: AD2 vs Veh, p=0.0159, Mann-Whitney test; Hippocampus: AD2 vs Veh, p=0.0333, t test. (**c**) Quantification of Iba1 intensity relative to selected area in the cortex and hippocampus of *App*$^{NL-F/NL-F}$ mice inoculated with vehicle (n=5) or AD2 brain extract (n=5). Cortex: AD2 vs Veh, p=0.0003; Hippocampus: AD2 vs Veh, p=0.0221, t test. (**d**) Quantification of microglia process by measuring the total length of branches per cell in the cortex and hippocampus of *App*$^{NL-F/NL-F}$ mice inoculated with vehicle (n=5) or AD2 brain extract (n=5). Cortex: AD2 vs Veh, p = 0.0427, t test. (**e**) The average number of Iba1-positive microglia associated with each plaque in the cortex and hippocampus of *App*$^{NL-F/NL-F}$ mice inoculated with vehicle (n=5) or AD2 brain extract (n=5). Cortex: AD2 vs Veh, p=0.0023; Hippocampus: AD2 vs Veh, p=0.0030, t test. Significant differences are denoted as *p<0.05, **p<0.01 and ***p<0.001. n.s. denotes not significant. Values are shown as mean ± SEM.

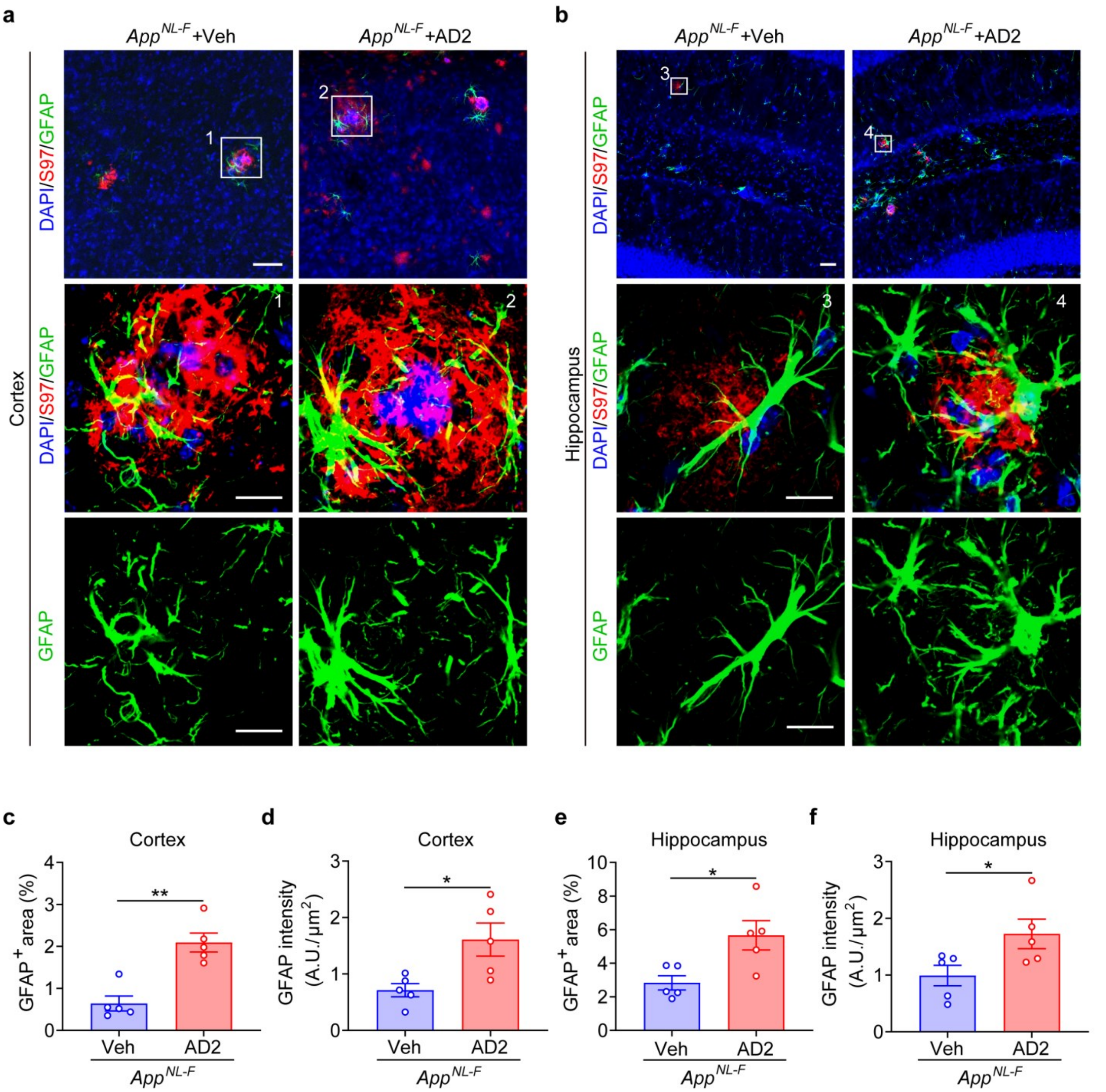

Supplementary Figure 5. Inoculation of *App*$^{NL-F/NL-F}$ mice with AD2 *S extract* induces astrocytosis.

(**a, b**) Representative images showing immunofluorescence staining of GFAP-positive astrocyte (green) and Aβ (S97, red), together with DAPI (blue) for cell nuclei in the cortex and hippocampus. Boxed regions in upper panels (scale bar: 40 µm) are numbered and shown with a higher magnification in lower panels (scale bar: 10 µm) and illustrate the astrocytosis associated with

amyloid plaques. (**c**) Quantification of the percentage of GFAP-positive astrocyte area in the cortex of $App^{NL\text{-}F/NL\text{-}F}$ mice inoculated with vehicle (n=5) or AD2 brain extract (n=5). AD2 vs Veh, p=0.0079, Mann-Whitney test. (**d**) Quantification of the GFAP intensity relative to selected area in the cortex of $App^{NL\text{-}F/NL\text{-}F}$ mice inoculated with vehicle (n=5) or AD2 brain extract (n=5). AD2 vs Veh, p=0.0215, t test. (**e**) Quantification of the percentage of GFAP-positive astrocyte area in the hippocampus of $App^{NL\text{-}F/NL\text{-}F}$ mice inoculated with vehicle (n=5) or AD2 brain extract (n=5). AD2 vs Veh, p=0.0193, t test. (**f**) Quantification of the GFAP intensity relative to selected area in the hippocampus of $App^{NL\text{-}F/NL\text{-}F}$ mice inoculated with vehicle (n=5) or AD2 brain extract (n=5). AD2 vs Veh, p=0.0495, t test. Significant differences are denoted as *$p<0.05$ and **$p<0.01$. Values are shown as mean ± SEM.

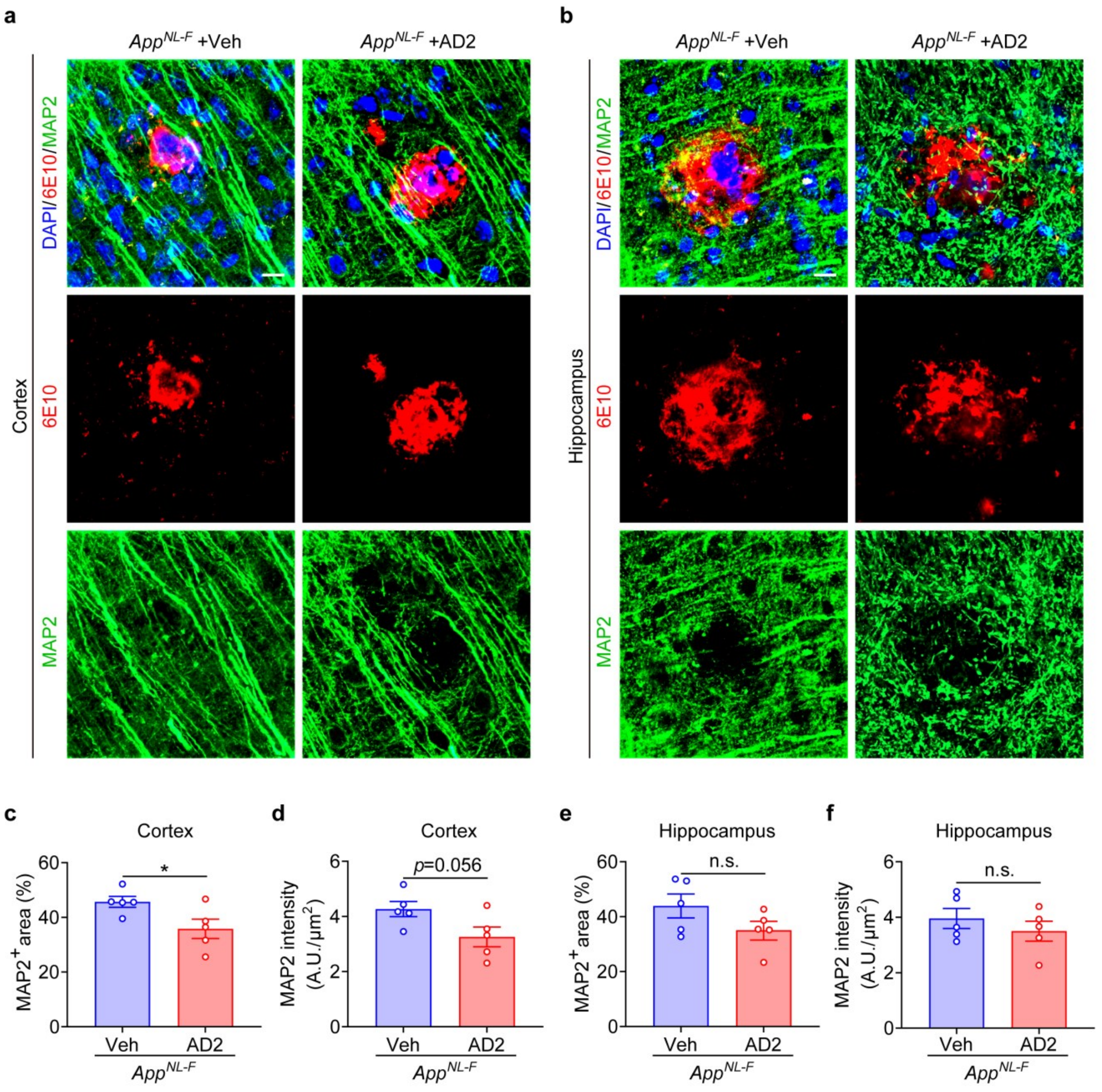

Supplementary Figure 6. Inoculation of *App*$^{NL\text{-}F/NL\text{-}F}$ mice with bioactive AD2 *S extract* tends to induce neuronal dystrophy. (**a, b**) Representative images showing immunofluorescence staining of dendritic marker MAP2 (green) and Aβ (6E10, red), together with DAPI (blue) for cell nuclei in the cortex and hippocampus. Scale bar: 10 μm. (**c**) Quantification of the percentage of MAP2-positive area in the cortex of *App*$^{NL\text{-}F/NL\text{-}F}$ mice inoculated with vehicle (n=5) or AD2 brain extract (n=5). AD2 vs Veh, p=0.0409, t test. (**d**) Quantification of the MAP2 intensity relative to selected area in the cortex of *App*$^{NL\text{-}F/NL\text{-}F}$ mice inoculated with vehicle (n=5) or AD2 brain extract (n=5).

AD2 vs Veh, *p*=0.0562, *t* test. (**e**) Quantification of the percentage of MAP2-positive area in the hippocampus of *App*$^{NL\text{-}F/NL\text{-}F}$ mice inoculated with vehicle (n=5) or AD2 brain extract (n=5). (**f**) Quantification of the MAP2 intensity relative to selected area in the hippocampus of *App*$^{NL\text{-}F/NL\text{-}F}$ mice inoculated with vehicle (n=5) or AD2 brain extract (n=5). Significant differences are denoted as $*p<0.05$. n.s. denotes not significant. Values are shown as mean ± SEM.

Supplementary Figure 7

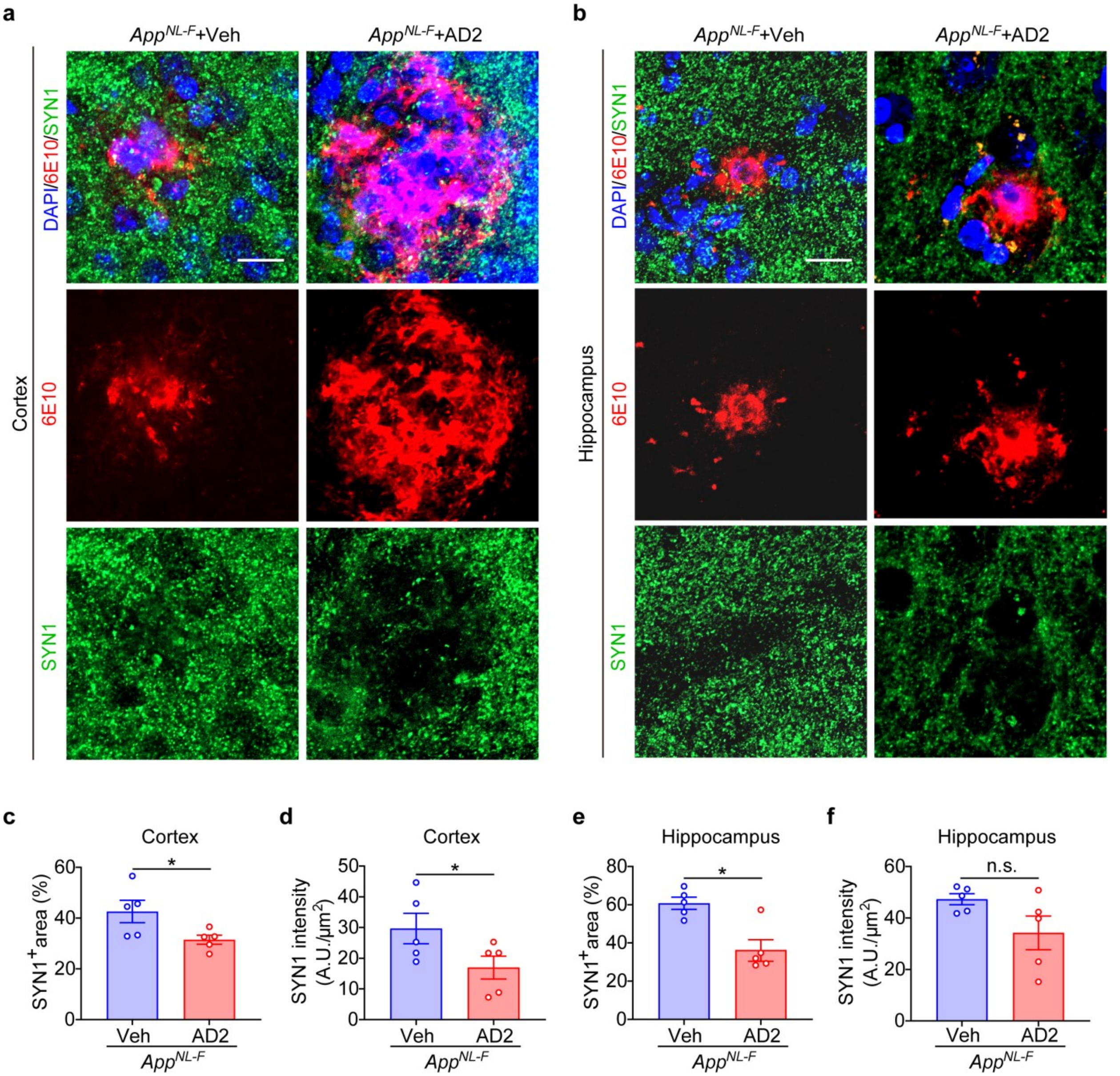

Supplementary Figure 7. Inoculation of *App*$^{NL\text{-}F/NL\text{-}F}$ mice with bioactive AD2 *S extract* induces synaptic loss. (**a, b**) Representative images showing immunofluorescence staining of synaptic marker SYN1 (green) and Aβ (6E10, red), together with DAPI (blue) for cell nuclei in the cortex and hippocampus. Scale bar: 10 μm. (**c**) Quantification of the percentage of SYN1-positive area in the cortex of *App*$^{NL\text{-}F/NL\text{-}F}$ mice inoculated with vehicle (n=5) or AD2 brain extract (n=5). AD2 vs

Veh, *p*=0.0475, *t* test. (**d**) Quantification of the SYN1 intensity relative to selected area in the cortex of *App*$^{NL\text{-}F/NL\text{-}F}$ mice inoculated with vehicle (n=5) or AD2 brain extract (n=5). AD2 vs Veh, *p*=0.0487, *t* test. (**e**) Quantification of the percentage of SYN1-positive area in the hippocampus of *App*$^{NL\text{-}F/NL\text{-}F}$ mice inoculated with vehicle (n=5) or AD2 brain extract (n=5). AD2 vs Veh, *p*=0.0317, Mann-Whitney test. (**f**) Quantification of the SYN1 intensity relative to selected area in the hippocampus of *App*$^{NL\text{-}F/NL\text{-}F}$ mice inoculated with vehicle (n=5) or AD2 brain extract (n=5). Significant differences are denoted as *p<0.05. n.s. denotes not significant. Values are shown as mean ± SEM.
